# On the Role of CD8^+^ T Cells in Determining Recovery Time from Influenza Virus Infection

**DOI:** 10.3389/fimmu.2016.00611

**Published:** 2016-12-20

**Authors:** Pengxing Cao, Zhongfang Wang, Ada W. C. Yan, Jodie McVernon, Jianqing Xu, Jane M. Heffernan, Katherine Kedzierska, James M. McCaw

**Affiliations:** ^1^School of Mathematics and Statistics, The University of Melbourne, Melbourne, VIC, Australia; ^2^Department of Microbiology and Immunology, The Peter Doherty Institute for Infection and Immunity, The University of Melbourne and Royal Melbourne Hospital, Melbourne, VIC, Australia; ^3^Shanghai Public Health Clinical Center, Key Laboratory of Medical Molecular Virology of Ministry of Education/Health, Shanghai Medical College, Institutes of Biomedical Sciences, Fudan University, Shanghai, China; ^4^Doherty Epidemiology, The Peter Doherty Institute for Infection and Immunity, The University of Melbourne and Royal Melbourne Hospital, Melbourne, VIC, Australia; ^5^Centre for Epidemiology and Biostatistics, Melbourne School of Population and Global Health, The University of Melbourne, Melbourne, VIC, Australia; ^6^Modelling and Simulation, Infection and Immunity Theme, Murdoch Childrens Research Institute, The Royal Children’s Hospital, Melbourne, VIC, Australia; ^7^Modelling Infection and Immunity Lab, Centre for Disease Modelling, York Institute for Health Research, Mathematics and Statistics, York University, Toronto, ON, Canada

**Keywords:** influenza, viral dynamics, mathematical model, cellular immunity, recovery time

## Abstract

Myriad experiments have identified an important role for CD8^+^ T cell response mechanisms in determining recovery from influenza A virus infection. Animal models of influenza infection further implicate multiple elements of the immune response in defining the dynamical characteristics of viral infection. To date, influenza virus models, while capturing particular aspects of the natural infection history, have been unable to reproduce the full gamut of observed viral kinetic behavior in a single coherent framework. Here, we introduce a mathematical model of influenza viral dynamics incorporating innate, humoral, and cellular immune components and explore its properties with a particular emphasis on the role of cellular immunity. Calibrated against a range of murine data, our model is capable of recapitulating observed viral kinetics from a multitude of experiments. Importantly, the model predicts a robust exponential relationship between the level of effector CD8^+^ T cells and recovery time, whereby recovery time rapidly decreases to a fixed minimum recovery time with an increasing level of effector CD8^+^ T cells. We find support for this relationship in recent clinical data from influenza A (H7N9) hospitalized patients. The exponential relationship implies that people with a lower level of naive CD8^+^ T cells may receive significantly more benefit from induction of additional effector CD8^+^ T cells arising from immunological memory, itself established through either previous viral infection or T cell-based vaccines.

## 1 Introduction

Invasion of influenza virus into a host’s upper respiratory tract leads to infection of healthy epithelial cells and subsequent production of progeny virions (1). Infection also triggers a variety of immune responses. In the early stage of infection, a temporary non-specific response (innate immunity) contributes to the rapid control of viral growth, while in the late stage of infection, the adaptive immune response dominates viral clearance (2). The early immune response involves production of antiviral cytokines and cells, e.g., type 1 interferon (IFN) and natural killer cells (NK cells), and is independent of virus type (3–7). In the special case of a first infection in a naive host, the adaptive immune response, mediated by the differentiation of naive T cells and B cells and subsequent production of virus-specific T cells and antibodies (2, 8), leads to not only a prolonged killing of infected cells and virus but also the formation of memory cells that can generate a rapid immune response to secondary infection with the same virus (9, 10).

CD8^+^ T cells, which form a major component of adaptive immunity, play an important role in efficient viral clearance (11). However, available evidence suggests that they are unable to clear virus in the absence of antibodies (12, 13) except in hosts with a very high level of preexisting naive or memory CD8^+^ T cells (14–16). Some studies indicate that depletion of CD8^+^ T cells could decrease the viral clearance rate and thus prolong the duration of infection (17–20). Furthermore, a recent study of human influenza A (H7N9) hospitalized patients has implicated the number of effector CD8^+^ T cells as an important driver of the duration of infection (21). These diverse experimental and clinical data, sourced from a number of host species, indicate that timely activation and elevation of CD8^+^ T cell levels may play a major role in the rapid and successful clearance of influenza virus from the host. These observations motivate our modeling study of the role of CD8^+^ T cells in influenza virus clearance.

Viral dynamics models have been extensively applied to the investigation of the antiviral mechanisms of CD8^+^ T cell immunity against a range of pathogens, with major contributions for chronic infections such as HIV/SIV (22–27), HTLV-I (28), and chronic LCMV (29, 30). However, for acute infections such as measles (31) and influenza (32–42), highly dynamical interactions between the viral load and the immune response occur within a very short-time window, presenting new challenges for the development of models incorporating CD8^+^ T cell immunity.

Existing influenza viral dynamics models, introduced to study specific aspects of influenza infection, are limited in their ability to capture all major aspects of the natural history of infection, hindering their use in studying the role of CD8^+^ T cells in viral clearance. Some models show a severe depletion of target cells (i.e., healthy epithelial cells susceptible to viral infection) after viral infection (34, 36–38, 40). Depletion may be due to either infection or immune-mediated protection. Either way, these models are arguably incompatible with recent evidence that the host is susceptible to reinfection with a second strain of influenza, a short period following primary exposure (43). Furthermore, as reviewed by Dobrovolny et al. (39), target cell depletion in these models strongly limits viral expansion so that virus can be effectively controlled or cleared at early stage of infection even in the absence of adaptive immunity, which contradicts the experimental finding that influenza virus remains elevated in the absence of adaptive immune response (44). While a few models do avoid target cell depletion (32, 33), they assume either immediate replenishment of target cells (32) or a slow rate of viral invasion into target cells resulting in a much delayed peak of virus titer at day 5 postinfection (rather than the observed peak at day 2) (33). Moreover, models with missing or unspecified major immune components, e.g., no innate immunity (24, 25, 36, 38), no antibodies (24, 25, 33, 41, 42), or unspecified adaptive immunity (40), also indicate the need for further model development. For an in-depth review of the current viral dynamics literature on influenza, we refer the reader to the excellent article by Dobrovolny et al. (39).

In this article, we construct a within-host model of influenza viral dynamics in naive (i.e., previously unexposed) hosts that incorporates the major components of both innate and adaptive immunity and use it to investigate the role of CD8^+^ T cells in influenza viral clearance. The model is calibrated against a set of published murine data by Miao et al. (38) and is then validated through demonstration of its ability to qualitatively reproduce a range of published data from immune-knockout experiments (12, 13, 17, 18, 38, 44). By using the model, we find that the recovery time—defined to be the time when virus titer first drops below a chosen threshold in the (deterministic) model—is negatively correlated with the level of effector CD8^+^ T cells in an approximately exponential manner. To the best of our knowledge, this relationship, with support from both H3N2-infected mice and H7N9-infected humans (21), has not been previously identified. The exponential relationship between CD8^+^ T cell level and recovery time is shown to be remarkably robust to variation in a number of key parameters, such as viral production rate, IFN production rate, delay of effector CD8^+^ T cell production, and the level of antibodies. Moreover, by using the model, we predict that people with a lower level of naive CD8^+^ T cells may receive significantly more benefit from induction of additional effector CD8^+^ T cells. Such production, arising from immunological memory, may be established through either previous viral infection or T cell-based vaccines.

## 2 Materials and Methods

### 2.1 The Model

**Figure 1:**
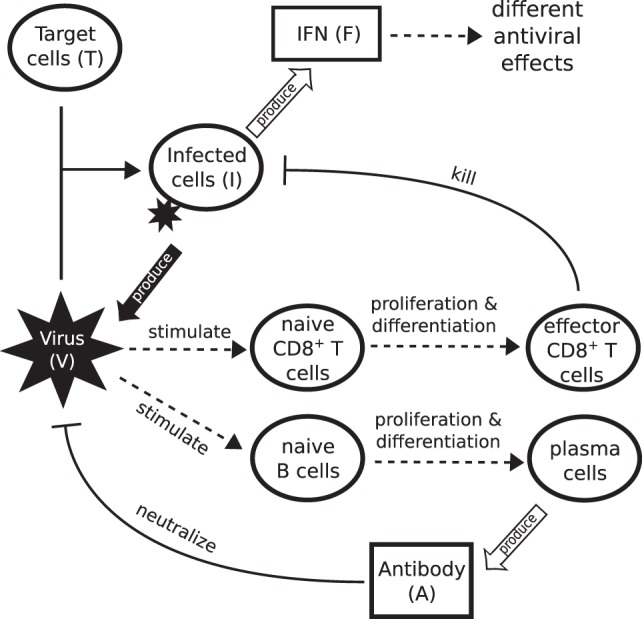
**Schematic diagram showing the major components of viral infection and the immune response**. Infection starts when virus binds to healthy epithelial cells (target cells). Infected cells release new virus and produce cytokines such as IFN. IFN is a major driver of innate immunity, responsible for effective control of rapid viral growth and expansion. Virus further stimulates naive CD8^+^ T cells and B cells to produce effector CD8^+^ T cells and antibodies, responsible for final clearance of virus.

The model of primary viral infection is a coupled system of ordinary and delay differential equations, consisting of three major components (see Figure 1 for a schematic diagram). Equations (1)–(3) describe the process of infection of target cells by influenza virus and are a major component in almost all models of viral dynamics in the literature. Equations (4) and (5) model IFN-mediated innate immunity (45, 46). Third, adaptive immunity including CD8^+^ T cells and B cell-produced antibodies for killing infected cells and neutralizing influenza virus, respectively, are described by equations (6)–(11).

(1) $$\frac{\mathrm{dV}}{\mathrm{dt}}=p_{V}I-\delta_{V}V-\kappa_{S}VA_{S}-\kappa_{L}{\mathrm{VA}}_{L}-\beta \mathrm{VT},$$

(2) $$\frac{\mathrm{dT}}{\mathrm{dt}}=g_{T}\left(T+R\right)\;\left(1-\frac{T+R+I}{T_{0}}\right)-\beta \prime \mathrm{VT}+\rho R-\phi \mathrm{FT},$$

(3) $$\frac{\mathrm{dI}}{\mathrm{dt}}=\beta \prime \mathrm{VT}-\delta_{I}I-\kappa_{N}\mathrm{IF}-\kappa_{E}\mathrm{IE},$$

(4) $$\frac{\mathrm{dF}}{\mathrm{dt}}=p_{F}I-\delta_{F}F,$$

(5) $$\frac{\mathrm{dR}}{\mathrm{dt}}=\phi \mathrm{FT}-\rho R,$$

(6) $$\frac{dC_{n}}{\mathrm{dt}}=-\beta_{\mathrm{Cn}}\;\left(\frac{V}{V+h_{C}}\right)\;C_{n},$$

(7) $$\frac{\mathrm{dE}}{\mathrm{dt}}=\beta_{\mathrm{Cn}}\;\left(\frac{V\left(t-\tau_{C}\right)}{V\left(t-\tau_{C}\right)+h_{C}}\right)\;C_{n}\left(t-\tau_{C}\right)e^{\left(p_{C}\tau_{C}\right)}-\delta_{E}E,$$

(8) $$\frac{dB_{n}}{\mathrm{dt}}=-\beta_{\mathrm{Bn}}\;\left(\frac{V}{V+h_{B}}\right)\;B_{n},$$

(9) $$\frac{\mathrm{dP}}{\mathrm{dt}}=\beta_{\mathrm{Bn}}\;\left(\frac{V\left(t-\tau_{B}\right)}{V\left(t-\tau_{B}\right)+h_{B}}\right)\;B_{n}\left(t-\tau_{B}\right)e^{\left(p_{B}\tau_{B}\right)}-\delta_{P}P,$$

(10) $$\frac{dA_{S}}{\mathrm{dt}}=p_{S}P-\delta_{S}A_{S},$$

(11) $$\frac{dA_{L}}{\mathrm{dt}}=p_{L}P-\delta_{L}A_{L}.$$

In further detail, equation (1) indicates that the change in viral load (*V*) is controlled by four factors: the production term (*p_V_I*) in which virions are produced by infected cells (*I*) at a rate of *p_V_* (37, 45, 47); the viral natural decay/clearance (*δ_V_V*) with a decay rate of *δ_V_*; the viral neutralization terms (*κ_S_VA_S_* and *κ_L_VA_L_*) by antibodies (both a short-lived antibody response *A_S_* driven by, e.g., IgM, and a longer-lived antibody response *A_L_* driven by, e.g., IgG and IgA (12, 38)), and a consumption term (*βVT*) due to binding to and infection of target cells (*T*). In equation (2), the term *g_T_*(*T* + *R*)(1 − (*T* + *R* + *I*)/*T*_0_) models logistic regrowth of the target cell pool (46). Both target cells (*T*) and resistant cells (*R*, those protected due to IFN-induced antiviral effect) can produce new target cells, with a net growth rate proportional to the severity of infection, 1 − (*T* + *R* + *I*)/*T*_0_ (i.e., the fraction of dead cells). *T*_0_ is the initial number of target cells and the maximum value for the target cell pool (34). Target cells (*T*) are consumed by virus (*V*) due to binding (*β*′*VT*), the same process as *βVT*. Note that *β* and β′ have different measurement units due to different units for viral load (*V*) and infected cells (*I*). As mentioned earlier, the innate response may trigger target cells (*T*) to become resistant (*R*) to virus at a rate *ϕFT*. Resistant cells lose protection at a rate ρ (45). This process also governs the evolution of virus-resistant cells (*R*) in equation (5).

Equation (3) describes the change of infected cells (*I*). They increase due to the infection of target cells by virus (β′*VT*) and die at a (basal) rate *δ_I_*. Two components of the immune response increase the rate of killing of infected cells. IFN-activated NK cells kill infected cells at a rate *κ_N_IF* (6, 45, 46, 48). Effector CD8^+^ T cells (*E*)—produced through differentiation from naive CD8^+^ T cells *C_n_* in equation (6)—kill at a rate *κ_E_IE*. Of note, our previous work has suggested that models of the innate response containing only IFN-induced resistance for target cells (state *R*; equation (5)), while able to maintain a population of healthy uninfected cells, still control viral kinetics through target cell depletion. While it remains possible that these models may be able to reproduce features of the viral reexposure data (43, 46), that work also demonstrated that inclusion of IFN-activated NK cells (term *κ_N_IF*) provides a natural explanation for the observed viral reexposure data.

Equation (4) models the innate response, as mediated by IFN (*F*). IFN is produced by infected cells at a rate *p_F_* and decays at a rate *δ_F_* (46).

Equation (6) models stimulation of naive CD8^+^ T cells (*C_n_*) into the proliferation/differentiation process by virus at a rate *β_Cn_V*/(*V* + *h_C_*), where *β_Cn_* is the maximum stimulation rate and *h_C_* indicates the viral load (titV) at which half of the stimulation rate is achieved. Note that this formulation does not capture the process of antigen presentation and CD8^+^ T cell activation, but rather is a simple way to establish the essential coupling between the viral load and the rate of CD8^+^ T cell activation in the model (49). In equation (7), the production of effector CD8^+^ T cells (*E*) is assumed to be an “advection flux” induced by a delayed virus stimulation of naive CD8^+^ T cells [the first term on the right-hand side of equation (7)]. The delayed variables, *V* (*t − τ_C_*) and *C_n_*(*t − τ_C_*), equal zero when *t < τ_C_*. The introduction of the delay *τ_C_* is to phenomenologically model the delay induced by both naive CD8^+^ T cell proliferation/differentiation and effector CD8^+^ T cell migration and localization to the site of infection for antiviral action (42, 50, 51). The delay also captures the experimental finding that naive CD8^+^ T cells continue to differentiate into effector T cells in the absence of ongoing antigenic stimulation (49, 52). The multiplication factor $e^{p_{C}\tau_{C}}$ indicates the number of effector CD8^+^ T cells produced from one naive CD8^+^ T cell, where *p_C_* is the average of effector CD8^+^ T cell production rate over the delay period *τ_C_*. The exponential form of the multiplication factor is derived based on the assumption that cell differentiation and proliferation follow a first-order advection–reaction equation. Effector CD8^+^ T cells decay at a rate *δ_E_*.

Similar to CD8^+^ T cells, equations (8) and (9) model the proliferation/differentiation of naive B cells, stimulated by virus presentation at rate *β_Bn_V*/(*V* + *h_B_*). Stimulation subsequently leads to the production of plasma B cells (*P*) after a delay *τ_B_*. The multiplication factor $e^{p_{B}\tau_{B}}$ indicates the number of plasma B cells produced from one naive B cell, where *p_B_* is the production rate. Plasma B cells secrete antibodies, which exhibit two types of profiles in terms of experimental observation: a short-lived profile (e.g., IgM lasting from about day 5 to day 20 postinfection) and a longer lived profile (e.g., IgG and IgA lasting weeks to months) (12, 38). These two antibody responses are modeled by equations (10) and (11), wherein different rates of production (*P_S_* and *P_L_*) and consumption (*δ_S_* and *δ_L_*) are assumed.

### 2.2 Model Parameters and Simulation

The model contains 11 equations and 30 parameters (see Table 1). This represents a serious challenge in terms of parameter estimation and clearly prevents a straightforward application of standard statistical techniques. To reduce uncertainty, a number of parameters were taken directly from the literature, as per the citations in Table 1. The rest were estimated (as indicated in Table 1) by calibrating the model against the published data from Miao et al. (38) who measured viral titer, CD8^+^ T cell counts, and IgM and IgG antibodies in laboratory mice (exhibiting a full immune response) over time during primary H3N2 A/Hong Kong/X31 influenza A virus infection (see Ref. (38) for a detailed description of the experiment). The approach to estimating the parameters based on Miao et al.’s data is provided in Supplementary Material, and the estimated parameter values are given in Table 1. Note that the data were presented in scatter plots in the original paper (38), while we presented the data here in mean ± SD at each data collection time point for a direct comparison with our mean-field mathematical model.

**Table 1:** **Model parameter values obtained by fitting the model to experimental data**.

Parmeter	Description	Value and unit	Reference
*V*_0_	Initial viral load	10^4^ [u_V_]	Estimated
*T*_0_	Initial number of epithelial cells in the URT	7 × 10^7^ cells	(53)
*g_T_*	Base growth rate of healthy cells	0.8 d^−1^	Fixed
*p_V_*	Viral production rate	210 [u_V_]cell^−1^ d^−1^	Estimated
*p_F_*	IFN production rate	10^−5^ [u_F_]cell^−1^ d^−1^	Estimated
*p_C_*	Naive CD8^+^ T cell proliferation rate	1.2 d^−1^	(32)
*p_B_*	Naive B cell proliferation rate	0.52 d^−1^	Estimated
*p_S_*	Short-lived antibody production rate	12 [u_A_]cell^−1^ d^−1^	Estimated
*p_L_*	Long-lived antibody production rate	4 [u_A_]cell^−1^ d^−1^	Estimated
*δ_V_*	Non-specific viral clearance rate	5 d^−1^	(47)
*δ_I_*	Non-specific death rate of infected cells	2 d^−1^	(32)
*δ_F_*	IFN degradation rate	2 d^−1^	(45)
*δ_E_*	Death rate of effector CD8^+^ T cells	0.57 d^−1^	(54)
*δ_P_*	Death rate of plasma B cells	0.5 d^−1^	Estimated
*δ_S_*	Short-lived antibody consumption rate	2 d^−1^	Estimated
*δ_L_*	Long-lived antibody consumption rate	0.015 d^−1^	Estimated
*β*	Rate of viral consumption by binding to target cells	5 × 10^−7^ cell^−1^ d^−1^	Estimated
*β*′	Rate of infection of target cells by virus	3 × 10^−8^ [u_V_]^−1^ d^−1^	Estimated
ϕ	Rate of conversion to virus-resistant state	0.33 [u_F_]^−1^ d^−1^	(45)
*ρ*	Rate of recovery from virus-resistant state	2.6 d^−1^	(45)
*κ_S_*	Rate of viral neutralization by short-lived antibodies	0.8 [u_A_]^−1^ d^−1^	Estimated
*κ_L_*	Rate of viral neutralization by long-lived antibodies	0.4 [u_A_]^−1^ d^−1^	Estimated
*κ_N_*	Killing rate of infected cells by IFN-activated NK cells	2.5 [u_F_]^−1^ d^−1^	(45)
*κ_E_*	Killing rate of infected cells by effector CD8^+^ T cells	5 × 10^−5^ cell^−1^ d^−1^	Estimated
*β_Cn_*	Maximum rate of stimulation of naive CD8^+^ T cells by virus	1 d^−1^	(9)
*β_Bn_*	Maximum rate of stimulation of naive B cells by virus	0.03 d^−1^	Estimated
*h_C_*	Half-maximal stimulating viral load for naive CD8^+^ T cells	10^4^ [u_V_]	Estimated
*h_B_*	Half-maximal stimulating viral load for naive B cells	10^4^ [u_V_]	Estimated
*τ_C_*	Delay for effector CD8^+^ T cell production	6 days	(51)
*τ_B_*	Delay for plasma B cell production	4 days	Estimated

*[*u_V_*], [*u_F_*], and [*u_A_*] represent the units of viral load, IFN, and antibodies respectively. [*u_V_*] and [*u_A_*] are measured as EID_50_/ml (50% egg infective dose) and pg/ml, consistent with the units of data. IFN is assumed to be a non-dimensionalized variable in the model, and therefore, [*u_F_*] can be ignored. Some parameters are obtained from the literature, and the rest are obtained by fitting the model to experimental data in the study by Miao et al. (38), except *g_T_*, which is of minor importance when considering a single infection and is thus fixed to reduce uncertainty*.

For model simulation, the initial condition is set to be (*V*, *T, I, F, R, C_n_, E, B_n_, P, A_S_, A_L_*) = (*V*_0_, *T*_0_, 0, 0, 0, 100, 0, 100, 0, 0, 0) unless otherwise specified. The initial target cell number (*T*_0_) was estimated by Petrie et al. (53). We estimate that of order 100 cells (resident in the spleen) are able to respond to viral infection (*C_n_*) (personal communication, N. LaGruta, Monash University, Australia). Note that 100 naive CD8^+^ T cells might underestimate the actual number of naive precursors that could respond to all the epitopes contained within the virus but does not qualitatively alter the model dynamics and predictions (see Section 3 where the naive CD8^+^ T cell number is varied between 0 and 200, where the upper bound is sufficient to show the model’s full range of behaviors). In the absence of further data, we also use this value for the initial naive B cell number (*B_n_*), but again this choice does not qualitatively alter the model predictions. The numerical method and code (implemented in MATLAB, version R2014b, the MathWorks, Natick, MA, USA) for solving the model are provided in Supplementary Material.

### 2.3 Analysis of Clinical Influenza A (H7N9) Data

Clinical influenza A (H7N9) patient data were used to test our model predictions on the relationship between CD8^+^ T cell number and recovery time. The data were collected from 12 surviving patients infected with H7N9 virus during the first wave of infection in China in 2013. (Raw data are provided in Data Sheet S1 in Supplementary Material; see the paper of Wang et al. (21) for details of data collection; this study was reviewed and approved by the SHAPHC Ethics Committee.) Note that the clinical data were scarce for some patients. For those patients, we have assumed that the available data are representative of the unobserved values in the neighboring time period. For each patient, we took the average IFNγ^+^ CD8^+^ T cell number in 10^6^ peripheral blood mononuclear cells (PBMC) for the period from day 8 to day 22 (or the recovery day if it comes earlier) postadmission as a measure of the effector CD8^+^ T cell level. This period was chosen *a priori* as it roughly matches the duration of the CD8^+^ T cell profile, and clinical samples were frequently collected in this period. The average CD8^+^ T cell count was given by the ratio of the total area under the data points (using trapezoidal integration) to the number of days from day 8 to day 22 (or the recovery day if it comes earlier). For those patients for whom samples at days 8 and/or 22 were missing, we specified the average CD8^+^ T cell level at the missing time point to be equal to the value from the nearest sampled time available.

## 3 Results

### 3.1 Model Properties and Reproduction of Published Experimental Data

**Figure 2:**
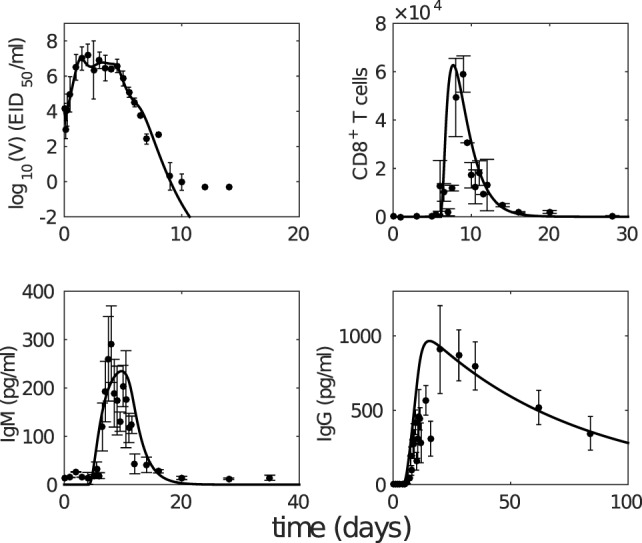
**The model with estimated parameters (solid curves) captures the murine data from the study by Miao et al. (38)**. The data are shown as error bars (mean ± SD). Note that due to the limit of detection for the viral load (occurring after 10 days postinfection as seen in viral load data), the last three data points in the upper left panel were not taken into consideration for model fitting.

**Figure 3:**
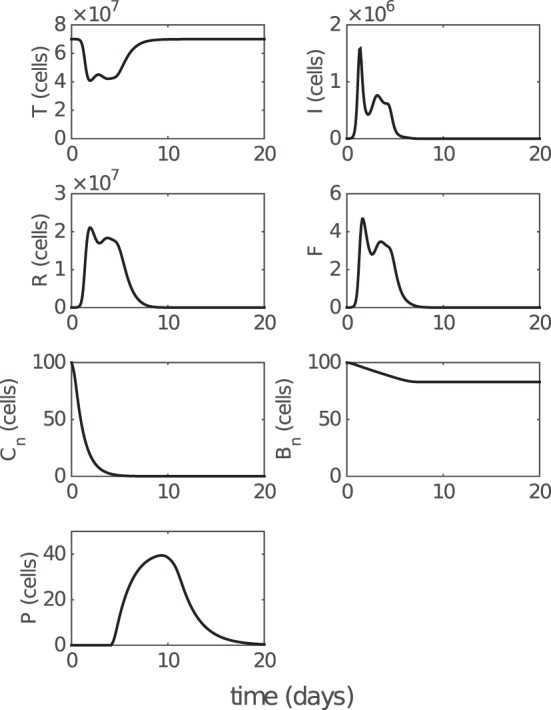
**Model solution for non-fitted variables**. *T, I*, and *R* represent the numbers of target cells, infected cells, and resistant cells, respectively. *F* represents the level of IFN (a dimensionless variable). *C_n_* and *B_n_* represent the numbers of naive CD8^+^ T cells and naive B cells, respectively. *P* represents the number of antibody-secreting plasma cells. Parameter values used to generate the results are given in Table 1. Note that model solutions for fitted variables are shown in Figure 2.

We first analyze the model behavior to establish a clear understanding of the model dynamics. Figure 2 shows solutions (time series) for the model compartments (viral load, CD8^+^ T cells, and IgM and IgG antibody) calibrated against the murine data from the study by Miao et al. (38). Solutions for the remaining model compartments are shown in Figure 3. The model (with both innate and adaptive components active) prevents the depletion of target cells (see Figure 3 wherein over 50% of target cells remain during infection) and results in a minor loss of just 10–20% of healthy epithelial cells (i.e., the sum of target cells (*T*) and virus-resistant cells (*R*); see Figure S1 in Supplementary Material). We note the important difference between prevention of target cell depletion on the one hand and maintenance of healthy cells on the other hand. To be compatible with heterologous reexposure studies (43, 46), a model must not only maintain the population of healthy cells (as many of the aforementioned models do) but must also prevent depletion of target cells to enable infection on rapid reexposure. Otherwise, if *T* is driven low and *R* high, while the healthy cell population will be maintained, infection on reexposure may still be blocked. In our model, the primary driver for the maintenance of the target cell pool during acute viral infection is a timely activation of the innate immune response (Figure S2 in Supplementary Material), indicating that our model improves upon previous models where viral clearance was only achieved through depletion of target cells (a typical solution shown in Figure S2B in Supplementary Material).

**Figure 4:**
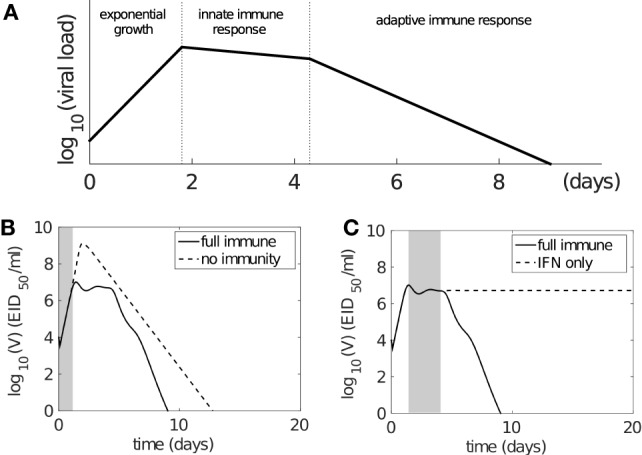
**The model solution exhibits three-phase behavior following influenza virus infection**. **(A)** Schematic representation of three phases of behavior based on involvement of immune responses. Following infection, virus first undergoes rapid exponential growth before the innate immune response is activated (on day 2). Innate immunity controls rapid viral expansion to form a plateau in viral load. Adaptive immunity starts to take effect 4–6 days postinfection and is responsible for viral clearance. **(B,C)** demonstrate that the model dynamics follow the three-phase theory. Viral kinetics in the presence of a full immune response is shown by the solid line **(B,C)**. **(B)** The dashed line shows viral kinetics in the complete absence of immunity (innate and adaptive; by letting *p_F_* = 0 and *β_Cn_* = *β_Bn_* = 0 in the model). The trajectories overlap prior to the activation of the innate response, before diverging due to target cell depletion. The shaded region highlights the first phase (exponential growth). **(C)** The dashed line shows viral kinetics in the absence of adaptive immunity (by letting *β_Cn_* = *β_Bn_* = 0; innate immunity remains active). The trajectories overlap prior to the activation of the adaptive response. The shaded region highlights the second phase (innate response). Note: changes in model parameters shifts where the three phases occur, but does not alter the underlying three-phase structure, i.e., existence of the three phases is robust to variation in parameters (see Supplementary Material and Figure S3 in Supplementary Material in particular).

The modeled viral dynamics exhibits three phases, each dominated by the involvement of different elements of the immune responses (Figure 4). Immediately following infection (0–2 days postinfection) and prior to the activation of the innate (and adaptive) immune responses, virus undergoes a rapid exponential growth (Figure 4A). In the second phase (2–5 days postinfection), the innate immune response successfully limits viral growth (Figure 4A). In the third phase (4–6 days postinfection), adaptive immunity (antibodies and CD8^+^ T cells) is activated and viral load decreases rapidly, achieving clearance. Figures 4B,C demonstrate the dominance of the different immune mechanisms at different phases. In Figure 4B, models with and without immunity are indistinguishable until day 2 (shaded region), before diverging dramatically when the innate and then adaptive immune responses influence the dynamics. In Figure 4C, models with and without an adaptive response only diverge at around day 4 as the adaptive response becomes active. We have further shown that this three-phase property is a robust feature of the model, emergent from its mathematical structure and not a property of fine tuning of parameters (see Figure S3 in Supplementary Material). Importantly, it clearly dissects the periods and effect of innate immunity, extending on previous studies of viral infection phases where the innate immune response was either ambiguous or ignored (12, 38, 55).

**Figure 5:**
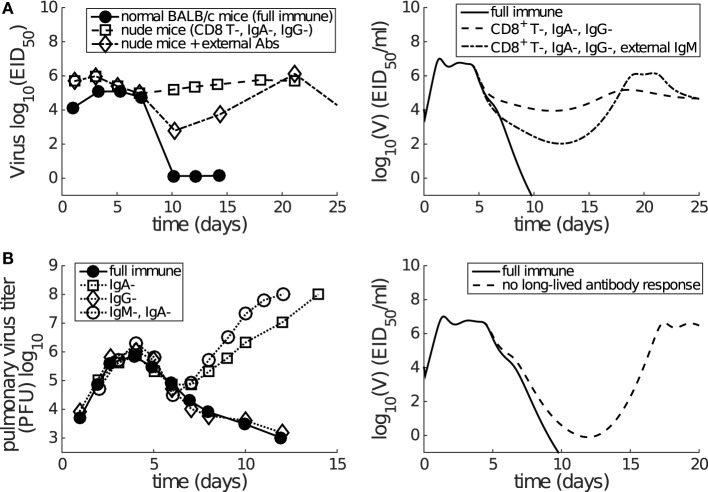
**Consistency between mice data (left panels) and model results (right panels) shows that short-lived antibody response (e.g., IgM) is only capable of generating a transient decrease in viral load while long-lived antibody response (e.g., IgA) plays a more dominant role in late-phase viral clearance**. **(A)** Data are from the paper of Kris et al. (44). Normal or nude BALB/c mice were infected with H3N2 virus. External antibodies were given at day 5 and had waned by about day 21. The model simulation mimics the passive antibody input by introducing an extra amount of IgM (in addition to antigen-stimulated IgM), whose time course faithfully follows the experimental measurement (see Figure 2A in the paper of Kris et al. (44)). “CD8^+^ T-, IgA-, IgG-” was modeled by letting *β_Cn_* = 0 and *p_L_* = 0. “External IgM” (in addition to the IgM produced by plasma cells) was modeled by adding a new term −*κ_S_VA_e_*(*t*) to equation (1), where *A_e_*(*t*) follows a piecewise function *A_e_*(*t*) = 0 for *t* < 5, *A_e_*(*t*) = 100(*t* − 5) for *t* ∈ [5, 7], *A_e_*(*t*) = 200 − 14(*t* − 7) for *t* ∈ [7, 21], and *A_e_*(*t*) = 0 for *t* ≥ 21. **(B)** Data are from the paper of Iwasaki and Nozima (12). The data indicate that the long-lasting IgA response, but not the long-lasting IgG response or the short-lasting IgM response, is necessary for successful viral clearance. “No long-lived antibody response” was modeled by letting *p_L_* = 0. Note that Miao et al. only measured IgM and IgG, but not IgA. As such, our model’s long-lived antibody response was calibrated against IgG kinetics (see Figure 2). Therefore, we emphasize that we can only investigate the relative contributions of short-lived and long-lived antibodies.

As reviewed by Dobrovolny et al. (39), a number of *in vivo* studies have been performed to dissect the contributions of CD8^+^ T cells and antibodies (12, 17, 18, 44, 56). We use the findings of these studies to validate our model, by testing how well it is able to reproduce the experimental findings (without any further adjustment to parameters). Although the determination of the role of CD8^+^ T cells is often hindered by co-inhibition of both CD8^+^ T cells and the long-lived antibody response (e.g., using nude mice), it is consistently observed that antibodies play a dominant role in final viral clearance, while CD8^+^ T cells are primarily responsible for the timely killing of infected cells and so indirectly contribute to an increased rate of removal of free virus toward the end of infection (13, 17, 18). Furthermore, experimental data demonstrate that a long-lived antibody response is crucial for achieving complete viral clearance, while short-lived antibodies are only capable of driving a transient decrease in viral load (12, 44). We find that our model (with parameters calibrated against Miao et al.’s data (38)) is able to reproduce these observations:

- Virus can rebound in the absence of long-lived antibody response (see Figure 5; Figure S4 in Supplementary Material).
- Both the CD8^+^ T cell response and short-lived antibody response only facilitate a faster viral clearance and are incapable of achieving clearance in the absence of long-lived antibody response (see Figure 5; Figure S4 in Supplementary Material).
- A lower level of CD8^+^ T cells (modulated by a decreased level of initial naive CD8^+^ T cells, *C_n_*) significantly prolongs the viral clearance (see Figure S4 in Supplementary Material).

In addition, the model also predicts a rapid depletion of naive CD8^+^ T cells after primary infection (see Figure 3), which represents a full recruitment of naive CD8^+^ T cell precursors. This result may be associated with the experimental evidence suggesting a strong correlation between the naive CD8^+^ T cell precursor frequencies and effector CD8^+^ T cell magnitudes for different pMHC-specific T cell populations (57). Note that in Figure 5, no adjustments to the model (e.g., to the vertical scale) were made; its behavior is completely determined by the calibration to the aforementioned murine data (38), and so these findings represent a (successful) prediction of the model.

In summary, we have demonstrated that our model—with parameters calibrated against murine data (38)—exhibits three important phases characterized by the involvement of various immune responses. Advancing on previous models, our model does not rely on target cell depletion and successfully reproduces a multitude of behavior from knockout experiments where particular components of the adaptive immune response were removed. This provides us with a well-tested platform, in which all major components of the immune response have been included and tested, with which to now make predictions on the effect of the cellular adaptive response on viral clearance.

### 3.2 Dependence of the Recovery Time on the Level of Effector CD8^+^ T Cells

Having established that our model is (from a structural point of view) biologically plausible and that our parameterization is capable of reproducing varied experimental data under different immune conditions (i.e., knockout experiments), we now study how the cellular adaptive response influences viral kinetics in detail. We focus on the key clinical outcome of recovery time, defined in the model as the time when viral titer first falls below 1 EID_50_/ml, the minimum value detected in relevant experiments (e.g., Figure 2).

**Figure 6:**
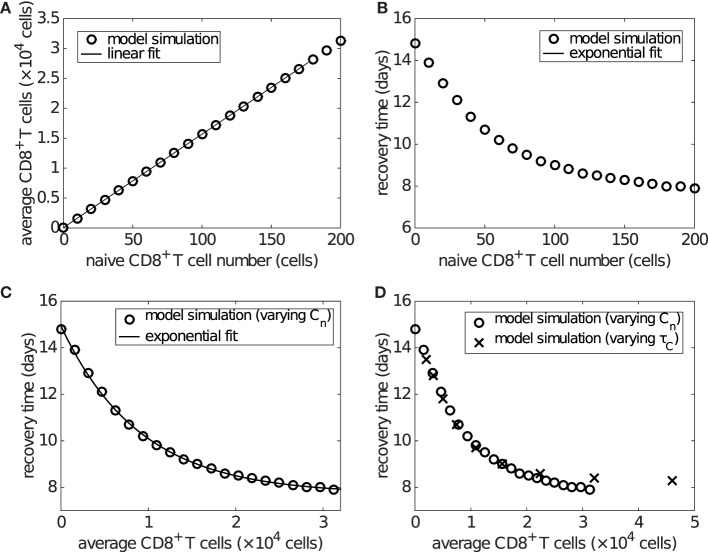
**The level of effector CD8^+^ T cells plays an important role in determining recovery time**. Recovery time is defined to be the time when viral load falls to 1 EID_50_/ml. **(A)** shows that the average effector CD8^+^ T cell number over days 6–20 is linearly related to the naive CD8^+^ T cell number (i.e., *C_n_*(0)). **(B)** shows that the recovery time is approximately exponentially related to the initial naive CD8^+^ T cell number. Combined, these results give **(C)**, wherein an approximately exponential relationship is observed between the average CD8^+^ T cell number and recovery time, both of which are experimentally measurable. Note that the exponential/linear fits shown in the figures are not generated by the viral dynamics model but are used to indicate the trends (evident visually) in the model’s behavior. **(D)** shows that varying the delay *τ_C_* (in a similar way to that shown in Figure S5 in Supplementary Material), rather than the naive CD8^+^ T cell number, does not alter the exponential relationship. In **(D)**, the crosses represent the results of varying *τ_C_* and the empty circles are the same as those in **(C)** for comparison.

Time series of the viral load show that the recovery time decreases as the initial naive CD8^+^ T cell number (*C_n_*) increases (Figure S4 in Supplementary Material). With that in mind, we now examine how recovery time is associated with the clinically relevant measure of effector CD8^+^ T cell level during viral infection. With an increasing initial level of naive CD8^+^ T cells, the average level of effector CD8^+^ T cells over days 6–20 increases linearly (Figure 6A), while the recovery time decreases in an approximately exponential manner (Figure 6B). Combining these two effects gives rise to an approximately exponential relation between the level of effector CD8^+^ T cells and recovery time (Figure 6C). Note that the exponential/linear fits shown in the figures are simply to aid in interpretation of the results. They are not generated by the viral dynamics model.

If varying the delay for naive CD8^+^ T cell activation and differentiation, *τ_C_*, while keeping the naive CD8^+^ T cell number fixed (at the default value of 100), we find that the average level of effector CD8^+^ T cells is exponentially related to the delay, while the recovery time is dependent on the delay in a piecewise linear manner (see Figure S5 in Supplementary Material). Nevertheless, the combination still leads to an approximately exponential relationship between the level of effector CD8^+^ T cells and recovery time (Figure S5C in Supplementary Material), which is almost identical to that of varying naive CD8^+^ T cells (Figure 6D). We also examine the sensitivity of the exponential relationship to other model parameters generally accepted to be important in influencing the major components of the system, such as the viral production rate *p_V_*, IFN production rate *p_F_*, and naive B cell number. We find that the exponential relationship is robust to significant variation in all of these parameters (see Figures S6 and S7 in Supplementary Material; the result for varying naive B cell number is shown in the last section of Section 3). These results suggest that a higher level of effector CD8^+^ T cells is critical for early recovery, consistent with experimental findings (58).

**Figure 7:**
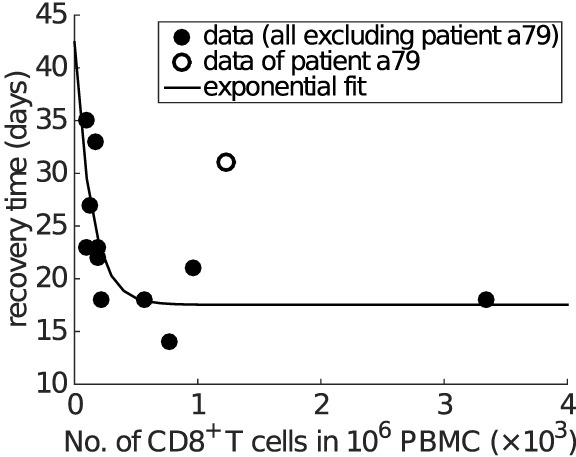
**Clinical influenza A (H7N9) data exhibit an exponential relationship between the average CD8^+^ T cell number and recovery time**. The x-axis is the average level of functional effector CD8^+^ T cells (i.e., IFNγ^+^ CD8^+^ T cells) over the period from day 8 to day 22 (or the recovery day if it comes earlier). Spearman’s rank correlation test indicates a significant negative correlation between the average CD8^+^ T cell numbers and recovery time (ρ = − 0.8368, *p* = 0.0013). Excluding one of the patients (No. a79 in Data Sheet S1 in Supplementary Material; discussed in Section 4), all other data points (solid dots) are fitted by an offset exponential function *y* = 24.8755e^−0.0073^*^x^* + 17.5356, indicating that the best achievable recovery time for individuals with a high CD8^+^ T cell response is approximately 17.5356 days.

Finally, and perhaps surprisingly given our model has been calibrated purely on data from the mouse, a strikingly similar relationship as shown in Figure 6C is found in clinical data from influenza A (H7N9) virus-infected patients (Figure 7). Excluding one patient (No. a79 in Data Sheet S1 in Supplementary Material; the exclusion is considered further in Section 4), average IFNγ^+^ CD8^+^ T cells and recovery time are negatively correlated (Spearman’s ρ = − 0.8368, *p* = 0.0013) and well captured by an exponential fit with an estimated offset (see Figure 7 caption for details). The exponential relationship (observed in both model and data) has features of a rapid decay for relatively low/intermediate levels of effector CD8^+^ T cells and a strong saturation for relatively high CD8^+^ T levels, implying that even with a very high level of naive CD8^+^ T cells, recovery time cannot be reduced below a certain value (in this case, estimated to be approximately 17 days). Of note, the exponential relationship (i.e., the scale of CD8^+^ T cell level or recovery time) is only a qualitative one, as we have no way to determine the scaling between different x-axis measurement units, nor adjust for particular host and/or viral factors that differ between the two experiments (i.e., H3N2-infection in the mouse (38) versus H7N9 infection in humans (21)).

### 3.3 Dependence of the Recovery Time on the Level of Memory CD8^+^ T Cells

In addition to naive CD8^+^ T cells, memory CD8^+^ T cells (established through previous viral infection) may also significantly affect recovery time due to both their rapid activation on antigen stimulus and faster replication rate (54, 59–61). To study the role of memory CD8^+^ T cells, we must first extend our model. As we are only concerned with how the presence of memory CD8^+^T cells influences the dynamics, as opposed to the development of the memory response itself, the model is modified in a straightforward manner through addition of two additional equations that describe memory CD8^+^ T cell (*C_m_*) proliferation/differentiation:

(12) $$\frac{dC_{m}}{\mathrm{dt}}=-\beta_{\mathrm{Cm}}\;\left(\frac{V}{V+h_{\mathrm{Cm}}}\right)\;C_{m},$$

(13) $$\frac{dE_{m}}{\mathrm{dt}}=\beta_{\mathrm{Cm}}\;\left(\frac{V\left(t-\tau_{\mathrm{Cm}}\right)}{V\left(t-\tau_{\mathrm{Cm}}\right)+h_{\mathrm{Cm}}}\right)\;C_{m}\left(t-\tau_{\mathrm{Cm}}\right)e^{\left(p_{\mathrm{Cm}}\tau_{\mathrm{Cm}}\right)}\;-\delta_{E}E_{m}.$$

Accordingly, the term *κ_E_IE* in equation (3) is modified to *κ_E_I*(*E* + *E_m_*). The full model and details on the choice of the additional parameters are provided in Supplementary Material. Note that the model component, *C_m_*, may include different populations of memory CD8^+^ T cells, including those directly specific to the virus and those stimulated by a different virus but which provide cross-protection (62, 63).

**Figure 8:**
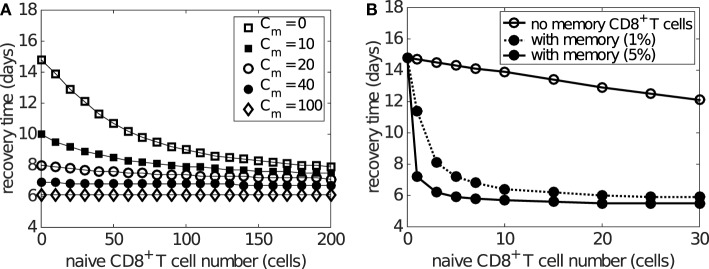
**Effects of naive and memory CD8^+^ T cells on viral clearance**. Recovery time is defined to be the time when the viral load falls to 1 EID_50_/ml. **(A)** demonstrates that varying the number of memory CD8^+^ T cells (*C_m_*) reduces the recovery time for any naive CD8^+^ T cell number (i.e. *C_n_*(0)). Note that saturation is observed for *C_m_* > 100 where the recovery time is about 6 days, independent of the naive cell numbers. **(B)** demonstrates how the presence of preexisting memory CD8^+^ T cells (solid dots) leads to a shorter recovery time when compared to the case where no memory CD8^+^ T cells are established (open circles). Note the time scale difference in **(A,B)**. This simulation is based on the assumption that the level of preexisting memory CD8^+^ T cells is assumed to be either 1 or 5% (as indicated in the legend) of the maximum effector CD8^+^ T cell number due to primary viral infection. The memory cell number (which is not shown in this figure) is about 30 times as many as the naive cell number shown in the figure, i.e., 30 naive cells result in about 900 memory cells before reinfection.

Figure 8A shows how the preexisting memory CD8^+^ T cell number (*C_m_*) changes the exponential relationship between naive CD8^+^ T cells and recovery time. Importantly, as the number of memory CD8^+^ T cells increases, the recovery time decreases for any level of naive CD8^+^ T cells and the exponential relationship remains. For patients with a relatively low level of naive CD8^+^ T cells (i.e., on the left of Figure 8A), the extent of reduction in the recovery time is greater than that for patients with a relatively high level of naive CD8^+^ T cells (i.e., on the right of Figure 8A). This suggests that people with a lower level of naive CD8^+^ T cells may benefit more through induction of memory CD8^+^ T cells, emphasizing the potential importance for taking prior population immunity into consideration when designing CD8^+^ T cell-based vaccines (64).

The above result is based on the assumption that the initial memory CD8^+^ T cell number upon reinfection is independent of the number of naive CD8^+^ T cells available during the previous infection. However, it has also been found that the stationary level of memory CD8^+^ T cells is usually maintained at about 5–10% of the maximum antigen-specific CD8^+^ T cell number during primary viral infection (8, 65). This indicates that people with a low naive CD8^+^ T cell number may also develop a low level of memory CD8^+^ T cells following infection. In consequence, such individuals may be relatively more susceptible to viral reinfection (66). This alternative and arguably more realistic relationship between the numbers of naive and memory CD8^+^ T cells is simulated in Figure 8B where memory CD8^+^ T cell levels are set to 5% of the maximum of the effector CD8^+^ T cell level. Results suggest that, on viral reinfection, preexisting memory CD8^+^ T cells are able to significantly improve recovery time except for hosts with a very low level of naive CD8^+^ T cells (Figure 8B). This is in accordance with the assumption that a smaller naive pool leads to a smaller memory pool and in turn a weaker shortening in recovery time. Although the model suggests that the failure of memory CD8^+^ T cells to protect the host is unlikely to be observed (because of the approximately 30-fold increase in the size of the memory pool relative to the naive pool), the failure range may be increased if the memory pool size is much smaller (modulated by, say, changing 5 to 1% in the model). Therefore, for people with a low naive CD8^+^ T cell number, the level of memory CD8^+^ T cells may be insufficient and prior infection may provide very limited benefit, further emphasizing the opportunity for novel vaccines that are able to induce a strong memory CD8^+^ T cell response to improve clinical outcomes.

### 3.4 Dependence of the Recovery Time on Antibody Level

**Figure 9:**
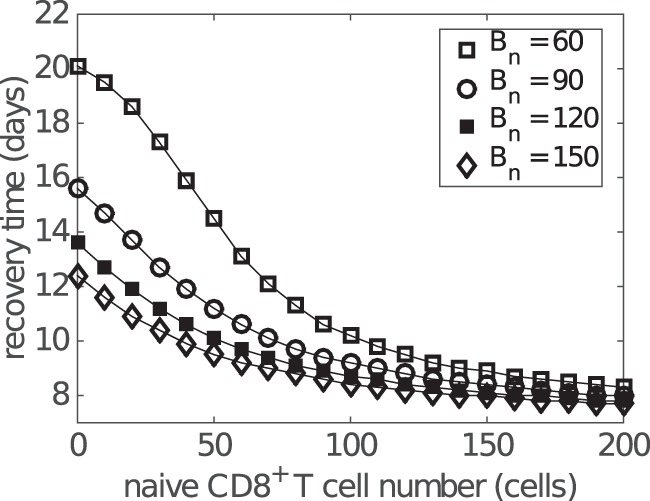
**Influence of antibody level on the relationship between the naive CD8^+^ T cell number and recovery time**. Recovery time is defined to be the time when viral load falls to 1 EID_50_/ml. Different antibody levels are simulated by varying the initial number of naive B cells (i.e., *B_n_* at *t* = 0).

Antibodies appear at a similar time as effector CD8^+^ T cells during influenza viral infection and may enhance the reduction in the recovery time in addition to CD8^+^ T cells. By varying the naive B cell number *B_n_* (as a convenient, but by no means unique, way to influence antibody level), we find that increasing the antibody level shortens the recovery time regardless of the initial naive CD8^+^ T cell number, leaving the exponential relation largely intact (Figure 9). A slight saturation occurs for the case in which levels of both naive B cells and CD8^+^ T cells are low. Moreover, variation in naive B cell number also results in a wider variation in recovery time for a lower naive CD8^+^ T cell level, suggesting that people with a lower level of naive CD8^+^ T cells may, once again, receive a more significant benefit (in terms of recovery time) through effective induction of an antibody response via vaccination.

## 4 Discussion

In this article, we have studied the role of CD8^+^ T cells in clearing influenza virus from the host using a viral dynamics model. The model was calibrated on a set of published murine data from the study by Miao et al. (38) and has been further shown to be able to reproduce a range of published data from experiments with different immune components knocked out. By avoiding target cell depletion, our model is also compatible with reinfection data (43), providing a strong platform on which to examine the role of CD8^+^ T cells in determining recovery time from infection. Our primary finding is that the time of recovery from influenza viral infection is negatively correlated with the level of effector CD8^+^ T cells in an approximately exponential manner. This robust property of infection has been identified from the model when calibrated against low pathogenic influenza A (H3N2) infection data in mice (38), but also observed in clinical case series of (severe) influenza A (H7N9) infection in humans (Figure 7) (21). Our findings, in conjunction with conclusions on the potential role for a T cell vaccine that stimulates and/or boosts the memory response, suggest new directions for research in both non-human species and further studies in humans on the association between CD8^+^ T cell levels and clinical outcomes. Further research, including detailed statistical fitting of our model to an extensive panel of infection data (as yet unavailable) from human and non-human species, is required to establish the generality of these relationships and provide quantitative insights for specific viruses in relevant hosts.

The non-linear relationship between effector CD8^+^ T cell level and recovery time may be useful in clinical treatment. The saturated property of the relation implies that a linear increase in the effector CD8^+^ T cell level may result in diminishing incremental improvements in patient recovery times. With evidence of a possible age-dependent loss of naive T cells (67–69), our model results imply that boosting the CD8^+^ T cell response via T cell vaccination may be particularly useful for those with insufficient naive CD8^+^ T cells. The population-level consequences of such boosting strategies, while beyond the scope of this work, have previously been considered by the authors (64).

We also investigated the effect of memory CD8^+^ T cell level on viral clearance and found unsurprisingly that a high preexisting level of memory CD8^+^ T cells was always beneficial. However, our results suggest that preexisting memory CD8^+^ T cells may be particularly beneficial for certain groups of people. For example, if the memory CD8^+^ T cell number induced by viral infection or vaccination is assumed to be relatively constant for everyone, people with less naive CD8^+^ T cells would benefit more on viral reinfection (see Figure 8A). On the other hand, if assuming preexisting memory CD8^+^ T cell number is positively correlated with the number of naive CD8^+^ T cells (simulated in Figure 8B), people with more naive CD8^+^ T cells would benefit more on viral reinfection. Emerging evidence suggests that the relationship between the level of memory CD8^+^ T cells and naive precursor frequencies is likely to be deeply complicated (57, 70–72). In that context, our model predictions emphasize the importance for further research in this area and the necessity to take prior population immunity into consideration when designing CD8^+^ T cell vaccines (64).

We modeled both short-lived and long-lived antibody responses. Experimental data and model predictions consistently show that the short-lived antibody response results in a temporary reduction in virus level, whereas the long-lived antibody response is responsible for complete viral clearance (Figure 5). We emphasize here that although the model is able to capture the observed short-lived and long-lived antibody responses (to study the virus–immune response interactions), it is not designed to investigate the mechanisms inducing different antibody responses. The observed difference in antibody decay profile may be a result of many factors including the life times of different antibody-secreting cell types (73, 74), different antibody life times (75), and antibody consumption through neutralizing free virions. Detailed study of these phenomena requires a more detailed model and associated data for parameter estimation and model validation and is thus left for future work. Similarly, CD4^+^ T cells are also known to perform a variety of functions in the development of immunity, such as facilitation of the formation and optimal recall of CD8^+^ T cells or even direct killing of infected cells during viral infection (9, 10, 76, 77). Their depletion due to, say, HIV infection has also been associated with more severe clinical outcomes following influenza infection (78). Some of the major functions of CD4^+^ T cells may be considered to be implicitly modeled through relevant parameters such as the rate of recall of memory CD8^+^ T cells (modeled by the delay *τ_Cm_*) in our extended model that includes memory CD8^+^ T cells. However, a detailed viral dynamics study of the role of CD4^+^ T cells in influenza infection, including in HIV-infected patients with depleted CD4^+^ T cells, remains an open and important challenge.

In a recent theoretical study, it was found that spatial heterogeneity in the T cell distribution may influence viral clearance (42). Resident CD8^+^ T cells in the lungs have a more direct and significant effect on timely viral clearance than do naive and memory pools resident in lymph nodes. Although this factor has been partially taken into consideration in our model by introducing a delay for naive/memory CD8^+^ T cells, lack of explicit modeling of the spatial dynamics limits a direct application of our model to investigate these spatial effects.

Finally, as noted in the results, one of the influenza A (H7N9)-infected patients (patient a79) was not included in our analysis of the clinical data (Figure 7). Although our model suggests some possibilities for the source of variation due to possible variation in parameter values, large variations in recovery time are only expected to occur for relatively low levels of naive CD8^+^ T cells, nominally incompatible with this patient’s moderate CD8^+^ T cell response but a relatively long recovery time. However, we note that IFNγ^+^ CD8^+^ T cell counts for this patient were only collected at days 10 and 23 and that the count at day 10 was particularly low and much lower than that at day 23 (see Data Sheet S1 in Supplementary Material). We suspect that delayed, rather than weakened, production (to at least day 10) of the IFNγ^+^ CD8^+^ T cell response in this patient substantially contributed to the observed delay in recovery. Further investigation of this patient’s clinical course and clinical samples is currently being undertaken.

## Author Contributions

PC, ZW, and JMM conceived the study. PC, AY, JH, and JMM developed the model. PC and AY carried out the analysis, with input from JM, JH, and JMM. ZW, JX, and KK provided the influenza A (H7N9) data and immunological insight. PC drafted the manuscript, with assistance from AY, JM, and JMM. All the authors gave final approval for publication.

## Conflict of Interest Statement

The authors declare that the research was conducted in the absence of any commercial or financial relationships that could be construed as a potential conflict of interest.
