# Supplementary material for: On the Role of CD8+ T Cells in Determining Recovery Time from Influenza Virus Infection
Source: Front Immunol. 2016 Dec 20;7:611. doi: 10.3389/fimmu.2016.00611 (PMC5167728; doi:10.3389/fimmu.2016.00611)
Supplement: Supplementary file 1 [file Presentation_1.PDF]

Supplementary Material for “On the role of $CD8^+$ T cells in determining recovery time from influenza virus infection”

Pengxing Cao[1], Zhongfang Wang[2,3], Ada W. C. Yan[1], Jodie McVernon[4,5], Jianqing Xu[3], Jane M. Heffernan[6], Katherine Kedzierska[2], and James M. McCaw*[1,4,5]

[1]School of Mathematics and Statistics, The University of Melbourne, Melbourne, Australia.
[2]Department of Microbiology and Immunology, University of Melbourne, at the Peter Doherty Institute for Infection and Immunity, Parkville, Victoria, Australia
[3]Shanghai Public Health Clinical Center and Institutes of Biomedical Sciences, Key Laboratory of Medical Molecular Virology of Ministry of Education/Health, Shanghai Medical College, Fudan University, Shanghai, China
[4]Centre for Epidemiology and Biostatistics, Melbourne School of Population and Global Health, The University of Melbourne, Melbourne, Australia.
[5]Modelling and Simulation, Infection and Immunity Theme, Murdoch Childrens Research Institute, The Royal Children’s Hospital, Parkville, Victoria, Australia.
[6]Modelling Infection and Immunity Lab, Centre for Disease Modelling, York Institute for Health Research, York University, Toronto, Ontario, Canada.

*Correspondence: jamesm@unimelb.edu.au

1 Numerical method for solving the model

The delay in activation of CD8$^+$ T cells and B cells directly results in the delay of production of effector CD8$^+$ T cells and antibody-producing cells. By a time-shift transform $t = t' + \tau_C$, Eq. 7 in the main text becomes

$$\frac{dE(t'+\tau_C)}{dt'} = \beta_{Cn}(\frac{V(t')}{V(t')+h_C})C_n(t')e^{(p_C\tau_C)} - \delta_E E(t'+\tau_C), \tag{S1}$$

where $t \geq 0$ and t' starts from $-\tau_C$. As defined in the main text, $V = 0$ for any negative time, i.e. $V(t') = 0$ for $-\tau_C < t' < 0$. Moreover, $E(t'+\tau_C) = 0$ for $\tau_C < t' < 0$. Thus, the equation is trivial for $t' \geq 0$. Therefore, for $t' \geq 0$, we replace t' back to t and Eq. S1 becomes

$$\frac{dE(t+\tau_C)}{dt} = \beta_{Cn}(\frac{V(t)}{V(t)+h_C})C_n(t)e^{(p_C\tau_C)} - \delta_E E(t+\tau_C), \tag{S2}$$

where $t \geq 0$. Similarly, Eqs. 9-11 in the main text can be changed to

$$\frac{dP(t+\tau_B)}{dt} = \beta_{Bn}(\frac{V(t)}{V(t)+h_B})B_n(t)e^{(p_B\tau_B)} - \delta_P P(t+\tau_B), \tag{S3}$$

$$\frac{dA_S(t+\tau_B)}{dt} = p_S P(t+\tau_B) - \delta_S A_S(t+\tau_B), \tag{S4}$$

$$\frac{dA_L(t+\tau_B)}{dt} = p_L P(t+\tau_B) - \delta_L A_L(t+\tau_B), \tag{S5}$$

In this way, the model presented in the main text is equivalent to the following model:

$$\frac{dV}{dt} = p_V I - \delta_V V - \kappa_S V A_S(t) - \kappa_L V A_L(t) - \beta V T, \tag{S6}$$

$$\frac{dT}{dt} = g_T(T+R)(1-\frac{T+R+I}{T_0}) - \beta' V T + \rho R - \phi F T, \tag{S7}$$

$$\frac{dI}{dt} = \beta' V T - \delta_I I - \kappa_N I F - \kappa_E I E(t), \tag{S8}$$

$$\frac{dF}{dt} = p_F I - \delta_F F, \tag{S9}$$

$$\frac{dR}{dt} = \phi F T - \rho R, \tag{S10}$$

$$\frac{dC_n}{dt} = -\beta_{Cn}(\frac{V}{V+h_C})C_n, \tag{S11}$$

$$\frac{dE(t+\tau_C)}{dt} = \beta_{Cn}(\frac{V}{V+h_C})C_n e^{(p_C\tau_C)} - \delta_E E(t+\tau_C), \tag{S12}$$

$$\frac{dB_n}{dt} = -\beta_{Bn}(\frac{V}{V+h_B})B_n, \tag{S13}$$

$$\frac{dP(t+\tau_B)}{dt} = \beta_{Bn}(\frac{V}{V+h_B})B_n e^{(p_B\tau_B)} - \delta_P P(t+\tau_B), \tag{S14}$$

$$\frac{dA_S(t+\tau_B)}{dt} = p_S P(t+\tau_B) - \delta_S A_S(t+\tau_B), \tag{S15}$$

$$\frac{dA_L(t+\tau_B)}{dt} = p_L P(t+\tau_B) - \delta_L A_L(t+\tau_B). \tag{S16}$$

For variables whose independent variable is not explicitly specified, they are all functions of t, i.e. V reads $V(t)$. This model avoids negative time and is solved by the following steps:

- Firstly choosing a time step size Δt and using it to discretise the time domain to be $t = 0, \Delta t, 2\Delta t, 3\Delta t, ..., N\Delta t$. The results in the main text are generated using $\Delta t = 0.1$ (day), the choice of which is based on the result that further decreasing Δt does not improve the solution (results not shown).

- Given initial condition

  $$(V,T,I,F,R,C_n,E,B_n,P,A_S,A_L) = (V_0, 7\times 10^7, 0, 0, 0, 100, 0, 100, 0, 0, 0),$$

  we solve the model iteratively. For iteration from $k\Delta t$ to $(k+1)\Delta t$, $A_S(t)$, $A_L(t)$ and $E(t)$ in Eqs. S6 and S8 are already known and thus treated as parameters. The system becomes an ODE system and can be easily solved by using a built-in ODE solver *ode15s* in MATLAB(R2014b) with default settings. All the variables at time $(k+1)\Delta t$ are then updated for use in the next iteration.

MATLAB code is provided below.

```
clear
dt=0.1; % time step size
time=0:dt:100;

% parameters
T0=7e+7;gT=0.8;pV=210;deltaV=5.0;
beta=5e-7;betap=3e-8;
deltaI=2;kappaN=2.5;kappaE=5e-5;
phi=0.33;rho=2.6;pF=1e-5;deltaF=2;
betaCn=1;betaBn=0.03;
kappaS=0.8;kappaL=0.4;
pC=1.2;pB=0.52;deltaE=0.57;deltaP=0.5;
pS=12;pL=4;deltaS=2;deltaL=0.015;
tauC=6;tauB=4;hC=1e+4;hB=1e+4;

% index indicating when delayed process starts
indC=round(tauC/dt+1); % for tauC
indB=round(tauB/dt+1); % for tauB

% variable vectors and initial conditions
V=zeros(1,length(time));V(1)=1e+4;
T=V;T(1)=7e+7;
I=T;I(1)=0;
R=I;
F=I;
Cn=100*ones(1,length(time));
Bn=100*ones(1,length(time));
E=zeros(1,indC+length(time));
P=I;
AS=zeros(1,indB+length(time));
AL=AS;

init=[V(1),T(1),I(1),R(1),F(1),Cn(1),E(1),Bn(1),P(1),AS(1),AL(1)]';
```

```
options = odeset('RelTol',1e-3,'AbsTol',1e-6);

for i=2:length(time)
    [~,Y]=ode15s(@ODEmodel,[0 dt],init,options,E(i),AL(i),AS(i),tauC,tauB,phi,rho,deltaF,gT,pF,pV,beta,betap,kappaN,deltaV,deltaI,betaCn,betaBn,kappaE,kappaS,pL,pS,deltaL,deltaS,deltaP,deltaE,pC,pB,kappaL,hC,hB,T0);

    V(i)=Y(end,1);T(i)=Y(end,2);I(i)=Y(end,3);R(i)=Y(end,4);
    F(i)=Y(end,5);Cn(i)=Y(end,6);Bn(i)=Y(end,8);P(i)=Y(end,9);
    E(indC+i)=Y(end,7);
    AS(indB+i)=Y(end,10);
    AL(indB+i)=Y(end,11);

    init=Y(end,:)'; % initial condition for next iteration
end
```

In the command of *ode15s*, a function “ODEmodel” is required and provided below.

```
function ynew=ODEmodel(~,y,E,AL,AS,tauC,tauB,phi,rho,deltaF,gT,pF,pV,beta,betap,kappaN,deltaV,deltaI,betaCn,betaBn,kappaE,kappaS,pL,pS,deltaL,deltaS,deltaP,deltaE,pC,pB,kappaL,hC,hB,T0)

% V: viral load
% T: target cell
% I: infected cell
% R: Resistant cell
% F: IFN
% Cn: naive CD8+ T cells
% E: effector CD8+ T cells
% Bn: naive B cells
% P: plasma B cells
% AS: short-lived antibodies
% AL: long-lived antibodies

% y=[V,T,I,R,F,Cn,E,Bn,P,AS,AL]

ynew=zeros(11,1);

ynew(1)=pV*y(3)-deltaV*y(1)-kappaS*y(1)*AS-kappaL*y(1)*AL-beta*y(1)*y(2);
ynew(2)=gT*(y(2)+y(4))*(1-(y(2)+y(3)+y(4))/T0)-betap*y(1)*y(2)+rho*y(4)-phi*y(2)*y(5);
ynew(3)=betap*y(1)*y(2)-deltaI*y(3)-kappaN*y(3)*y(5)-kappaE*y(3)*E;
ynew(4)=phi*y(2)*y(5)-rho*y(4);
ynew(5)=pF*y(3)-deltaF*y(5);
```

```
ynew(6)=-betaCn*y(1)/(y(1)+hC)*y(6);
ynew(7)=betaCn*y(1)/(y(1)+hC)*y(6)*exp(pC*tauC)-deltaE*y(7);
ynew(8)=-betaBn*y(1)/(y(1)+hB)*y(8);
ynew(9)=betaBn*y(1)/(y(1)+hB)*y(8)*exp(pB*tauB)-deltaP*y(9);
ynew(10)=pS*y(9)-deltaS*y(10);
ynew(11)=pL*y(9)-deltaL*y(11);
```

2 Details of fitting the model to data

The model contains 11 equations and 30 parameters (see Table 1 in the main text). This represents a serious challenge in terms of parameter estimation, and clearly prevents a straightforward application of standard statistical techniques. However, based on an extensive survey of the experimental literature, we have been able to identify plausible, but by no means unique, combinations of parameters that successfully explain the available data. A number of parameters were taken directly from the literature, as per the citations in Table 1. The rest (18 parameters) were estimated by calibrating the model to the published data from Miao *et al.* (1) who measured viral titre, $CD8^+$ T cell counts and IgM and IgG antibodies in laboratory mice (exhibiting a full immune response) over time during primary influenza H3N2 virus infection (shown in Fig. 2 in the main text). Note that the data were presented in scatter plots in the original paper (1), while we presented the data in Mean $\pm$ SD at each data collection time point (as shown in Fig. 2 in the main text) and fit our mean-field mathematical model to the means.

To obtain the set of parameters used for the main analysis from the experimental data in the paper of Miao *et al.* (1), we took the following approach:

- We first manually determined a set of the 18 parameters which produced a model solution that reasonably matched the experimental data shown in Fig. 2 in the main text. In detail, the main criteria include: 1) the viral load starts from about 10^4EID$_{50}$/ml, reaches a peak of about 10^7EID$_{50}$/ml at 1–2 days p.i., then declines rapidly from about 5 days p.i. (note that the last three data points were not considered due to the limit of detection); 2) the $CD8^+$ T cell count starts to increase rapidly at about 6 days p.i., reaches a peak of about 10^5–10^6 at 8–10 days p.i. and returns back to zero after about 20 days p.i.; 3) the IgM level starts to increase rapidly at 4–5 days p.i., reaches a peak of about 200–300 pg/ml at about 10 days p.i. and returns back to baseline after about 20 days p.i.; 4) the IgG level starts to increase rapidly at 4–5 days p.i., reaches a peak of about 800–1000 pg/ml at about 20 days p.i. and decays slowly. Given the high-dimensionality of the parameter space and limited experimental data, the procedure was essential in allowing us to identify a candidate parameter set which was not far from generating a local minimum in the following optimisation process.

- The candidate parameter set was then used as an initial estimate for optimization using MATLAB's built-in function *fmincon* with default settings. The target of optimization was to minimize the least-squares error (LSE):

$$L = L_V + L_E + L_M + L_G, \tag{S17}$$

  where the four LSE components for viral load, effector $CD8^+$ T cells, IgM and IgG were given respectively by

$$L_V = \sum_i [(V_{model}(t_i) - V_{data}(t_i)) w_V(t_i)/s_V]^2, \tag{S18}$$

$$L_E = \sum_i [(E_{model}(t_i) - E_{data}(t_i)) w_E(t_i)/s_E]^2, \tag{S19}$$

$$L_M = \sum_i [(M_{model}(t_i) - M_{data}(t_i)) w_M(t_i)/s_M]^2, \tag{S20}$$

$$L_G = \sum_i [(G_{model}(t_i) - G_{data}(t_i)) w_G(t_i)/s_G]^2. \tag{S21}$$

V_{model}, E_{model}, M_{model} and G_{model} indicate the model solution for variables V, E, A_S and A_L evaluated at time t_i respectively. V_{data}, E_{data}, M_{data} and G_{data} indicate the associated data. i is the index of the time point and the t_i may differ for the four components. $s_V = 10^7$, $s_E = 6 \times 10^4$, $s_M = 300$ and $s_G = 900$ were used to scale the errors to the same order of magnitude. Due to the fact that data were not collected at a fixed frequency (i.e. the time interval between adjacent data points is not constant), the errors at different time points were assigned different weights based on the length of time intervals between adjacent points. For example, if viral load was measured at time $t_i, (i = 0,1,2,...,k)$, the weight function $w_V(t_i)$ for interior points was given by

$$w_V(t_i) = \frac{t_{i+1} - t_{i-1}}{2(t_k - t_0)}, \tag{S22}$$

and for boundary points by

$$w_V(t_0) = \frac{t_1 - t_0}{2(t_k - t_0)} \qquad \text{and} \qquad w_V(t_k) = \frac{t_k - t_{k-1}}{2(t_k - t_0)}. \tag{S23}$$

This error weighting was used to weaken the domination of dense data points on model fits. It is evident that a lot of measurements were done within the first 10 days post-infection but only a few were performed after day 20 post-infection, in particular for IgG data (see Fig. 2 in the main text). We found that using equally weighted LSEs led to a model fit that manifestly failed to capture those sparse data points which we believe are equally, if not more important from a more biological perspective, in determining the IgG kinetics (see Fig. S8, compared with Fig. 2 in the main text).

- The parameter constraints when using *fmincon* were set to $V_0 \in [10^3, 10^5]$, $p_V \in [50, 500]$, $\beta \in [0,1]$, $\beta' \in [0,1]$, $p_F \in [0,1]$, $\kappa_S \in [0,2]$, $\kappa_L \in [0,1]$, $\kappa_E \in [0,1]$, $\beta_{Bn} \in [0,2]$, $p_B \in [0,1]$, $\delta_P \in [0,1]$, $\delta_S \in [0,5]$, $p_S \in [0,20]$, $p_L \in [0,20]$, $\delta_L \in [0,0.1]$, $\tau_B \in [3,7]$, $h_C \in [10^3, 10^5]$ and $h_B \in [10^3, 10^5]$.

- After obtaining a locally optimized solution for the candidate parameter set, we then checked the solution generated and evaluated its biological plausibility (based on the criteria mentioned above). This step was essential as given the over-specification of the model (in a statistical sense), it was possible for good fitting solutions to be identified by MATLAB's optimization algorithm, which were nonetheless biologically implausible. For example, oscillatory solutions for quantities such as IgG, while providing a "good-fit" to data, were not deemed acceptable on biological grounds (see Fig. S9 for such an example). If the optimised solution failed our (qualitative) evaluation, we returned to the first step and redetermined a new set of parameters as new initial estimates to be optimized using MATLAB.

This entire process was repeated to arrive at the default parameter set shown in Table 1 in the main text. To guarantee that the default parameter set was a good choice, we further randomly generated 10,000 sets of parameter samples near the default parameter set (within ±50% from the default values) and used them as initial estimates with *fmincon* to search for locally optimized solutions. Of these, 31 generated a better LSE but all failed to meet the criteria mentioned above (results not shown).

Fig. 2 in the main text shows how the model reproduces the key dynamic behavior shown in the data. We also show in the *Results* section in the main text, that the model behavior is robust to perturbation of model parameters and that model predictions are reasonably consistent with

a range of other experimental data, demonstrating the plausibility, if not uniqueness (of course), of the parameter set. We emphasize that, although the default parameter set is successful in reproducing a multitude of experimental observations (e.g. full immune, knockout, re-infection) as presented in the main text, we by no means claim that this parameter set is unique. It remains an open and challenging problem to reliably identify a biologically plausible and statistically identifiable solution for what is a highly complex system, where we are severely limited by available experimental data.

3 The model with memory CD8$^+$ T cells

Incorporating memory CD8$^+$ T cells into the model in the main text, we only make two changes. The first is adding two equations to describe the memory CD8$^+$ T cell (C_m) proliferation/differentiation, similar to Eqs. 6 and 7 in the main text

$$\frac{dC_m}{dt} = -\beta_{Cm}(\frac{V}{V+h_{Cm}})C_m, \tag{S24}$$

$$\frac{dE_m}{dt} = \beta_{Cm}(\frac{V(t-\tau_{Cm})}{V(t-\tau_{Cm})+h_{Cm}})C_m(t-\tau_{Cm})e^{(p_{Cm}\tau_{Cm})} - \delta_E E_m. \tag{S25}$$

Then we change the term $\kappa_E IE$ in Eq. 3 in the main text to $\kappa_E I(E+E_m)$. Hence, similar to the approach mentioned above that moving the delayed term from viral load to effector cells, we write down the model in an equivalent form,

$$\frac{dV}{dt} = p_V I - \delta_V V - \kappa_S V A_S(t) - \kappa_L V A_L(t) - \beta VT, \tag{S26}$$

$$\frac{dT}{dt} = g_T(T+R)(1-\frac{T+R+I}{T_0}) - \beta' VT + \rho R - \phi FT, \tag{S27}$$

$$\frac{dI}{dt} = \beta' VT - \delta_I I - \kappa_N IF - \kappa_E I[E(t)+E_m(t)], \tag{S28}$$

$$\frac{dF}{dt} = p_F I - \delta_F F, \tag{S29}$$

$$\frac{dR}{dt} = \phi FT - \rho R, \tag{S30}$$

$$\frac{dC_n}{dt} = -\beta_{Cn}(\frac{V}{V+h_C})C_n, \tag{S31}$$

$$\frac{dE(t+\tau_C)}{dt} = \beta_{Cn}(\frac{V}{V+h_C})C_n e^{(p_C\tau_C)} - \delta_E E(t+\tau_C), \tag{S32}$$

$$\frac{dB_n}{dt} = -\beta_{Bn}(\frac{V}{V+h_B})B_n, \tag{S33}$$

$$\frac{dP(t+\tau_B)}{dt} = \beta_{Bn}(\frac{V}{V+h_B})B_n e^{(p_B\tau_B)} - \delta_P P(t+\tau_B), \tag{S34}$$

$$\frac{dA_S(t+\tau_B)}{dt} = p_S P(t+\tau_B) - \delta_S A_S(t+\tau_B), \tag{S35}$$

$$\frac{dA_L(t+\tau_B)}{dt} = p_L P(t+\tau_B) - \delta_L A_L(t+\tau_B). \tag{S36}$$

$$\frac{dC_m}{dt} = -\beta_{Cm}(\frac{V}{V+h_{Cm}})C_m, \tag{S37}$$

$$\frac{dE_m(t+\tau_{Cm})}{dt} = \beta_{Cm}(\frac{V}{V+h_{Cm}})C_m e^{(p_{Cm}\tau_{Cm})} - \delta_E E_m(t+\tau_{Cm}). \tag{S38}$$

For variables whose independent variables are not explicitly specified, they are all functions of t, i.e. V reads $V(t)$. Memory CD8$^+$ T cells show a shorter delay and faster proliferation than naive CD8$^+$ T cells (2). The shortened delay may be caused by a shortened lag time to the first division and/or a reduced delay for effector cells migrating from the lymphatic compartment to the lung (2, 3, 4). The former reduction is about 15 hours (2) and the latter is less than about 12 hours (3). Thus, we choose $\tau_{Cm} = 5$ (days), correspond to a one day reduction in the delay time compared to the delay of naive cells ($\tau_C = 6$). Memory CD8$^+$ T cells show a higher division

rate and a lower loss rate than naive CD8$^+$ T cells (2), based on which the net production rate of effector cells for memory CD8$^+$ T cells is estimated to be about 1.5 times of that for naive cells. Thus,we choose $p_{Cm} = 1.5p_C = 1.8$ (day^{-1}). In the absence of data, we assume $\beta_{Cm} = \beta_C$ and $h_{Cm} = h_C$. The initial number of memory CD8$^+$ T cells is varied as specified in the main text or figures. We assume that the effector CD8$^+$ T cells produced by either naive or memory CD8$^+$ T cells are functionally identical (i.e. then have the same decay rate δ_E and killing rate κ_E). Note that we do not model the process of differentiation of effector CD8$^+$ T cells into memory cells but use a memory cell pool as an initial condition to simulate viral re-infection.

MATLAB code is provided below.

```
clear
dt=0.1; % time step size
time=0:dt:100;
% parameters
T0=7e+7;gT=0.8;pV=210;deltaV=5.0;
beta=5e-7;betap=3e-8;
deltaI=2;kappaN=2.5;kappaE=5e-5;
phi=0.33;rho=2.6;pF=1e-5;deltaF=2;
betaCn=1;betaBn=0.03;
kappaS=0.8;kappaL=0.4;
pC=1.2;pB=0.52;deltaE=0.57;deltaP=0.5;
pS=12;pL=4;deltaS=2;deltaL=0.015;
tauC=6;tauB=4;hC=1e+4;hB=1e+4;
betaCm=1;pCm=1.8;tauCm=5;

% index indicating when delayed process starts
indC=round(tauC/dt+1); % for tauC
indCm=round(tauCm/dt+1); % for tauCm
indB=round(tauB/dt+1); % for tauB

% variable vectors and initial conditions
V=zeros(1,length(time));V(1)=1e+1;
T=V;T(1)=7e+7;I=T;I(1)=0;R=I;F=I;
Cn=100*ones(1,length(time));
Bn=100*ones(1,length(time));
E=zeros(1,indC+length(time));
P=I;AS=zeros(1,indB+length(time));AL=AS;
Cm=5000*ones(1,length(time));
Em=zeros(1,indCm+length(time));

init=[V(1),T(1),I(1),R(1),F(1),Cn(1),E(1),Bn(1),P(1),AS(1),AL(1),
    Cm(1),Em(1)]';

options = odeset('RelTol',1e-3,'AbsTol',1e-6);

for i=2:length(time)
    [~,Y] = ode15s(@ODEmodel_with_memory,[0 dt],init,options,E(i),AL(i),AS(i),Em(i),tauC,tauB,phi,rho,deltaF,gT,pF,pV,
```

```
        beta, betap, kappaN, deltaV, deltaI, betaCn, betaBn, kappaE,
        kappaS, pL, pS, deltaL, deltaS, deltaP, deltaE, pC, pB, kappaL, hC
        , hB, T0, betaCm, pCm, tauCm);

    V(i)=Y(end,1);T(i)=Y(end,2);I(i)=Y(end,3);R(i)=Y(end,4);
    F(i)=Y(end,5);Cn(i)=Y(end,6);Bn(i)=Y(end,8);P(i)=Y(end,9);
    E(indC+i)=Y(end,7);
    AS(indB+i)=Y(end,10);AL(indB+i)=Y(end,11);
    Cm(i)=Y(end,12);Em(indCm+i)=Y(end,13);
    init=Y(end,:)'; % initial condition for next iteration
end
```

The function "ODEmodel_with_memory" is provided below.

```
function ynew=ODEmodel_New_with_memory(~,y,E,AL,AS,Em,tauC,
    tauB,phi,rho,deltaF,gT,pF,pV,beta,betap,kappaN,deltaV,deltaI
    ,betaCn,betaBn,kappaE,kappaS,pL,pS,deltaL,deltaS,deltaP,
    deltaE,pC,pB,kappaL,hC,hB,T0,betaCm,pCm,tauCm)

% V: viral load
% T: target cell
% I: infected cell
% R: Resistant cell
% F: IFN
% Cn: naive CD8+ T cells
% E:  effector CD8+ T cells
% Bn: naive B cells
% P:  plasma B cells
% AS: short-lived antibodies
% AL: long-lived antibodies
% Cm:  memory CD8+ T cells
% Em:  effector CD8+ T cells produced from memory cells

% y=[V,T,I,R,F,Cn,E,Bn,P,AS,AL,Cm,Em]

ynew=zeros(13,1);
ynew(1)=pV*y(3)-deltaV*y(1)-kappaS*y(1)*AS-kappaL*y(1)*AL-beta
    *y(1)*y(2);
ynew(2)=gT*(y(2)+y(4))*(1-(y(2)+y(3)+y(4))/T0)-betap*y(1)*y(2)
    +rho*y(4)-phi*y(2)*y(5);
ynew(3)=betap*y(1)*y(2)-deltaI*y(3)-kappaN*y(3)*y(5)-kappaE*y
    (3)*(E+Em);
ynew(4)=phi*y(2)*y(5)-rho*y(4);
ynew(5)=pF*y(3)-deltaF*y(5);
ynew(6)=-betaCn*y(1)./(y(1)+hC)*y(6);
ynew(7)=betaCn*y(1)./(y(1)+hC)*y(6)*exp(pC*tauC)-deltaE*y(7);
ynew(8)=-betaBn*y(1)./(y(1)+hB)*y(8);
ynew(9)=betaBn*y(1)./(y(1)+hB)*y(8)*exp(pB*tauB)-deltaP*y(9);
ynew(10)=pS*y(9)-deltaS*y(10);
```

```
ynew(11)=pL*y(9)-deltaL*y(11);
ynew(12)=-betaCm*y(1)./(y(1)+hC)*y(12);
ynew(13)=betaCm*y(1)./(y(1)+hC)*y(12)*exp(pCm*tauCm)-deltaE*y
    (13);
```

References

1. Miao H, Hollenbaugh JA, Zand MS, Holden-Wiltse J, Mosmann TR, Perelson AS, et al. Quantifying the early immune response and adaptive immune response kinetics in mice infected with influenza A virus. *J Virol* (2010) **84:**6687–6698. doi:10.1128/JVI.00266-10.

2. Veiga-Fernandes H, Walter U, Bourgeois C, McLean A, Rocha B. Response of naive and memory $CD8^+$ T cells to antigen stimulation *in vivo*. *Nat Immunol* (2000) **1(1):**47–53. doi:10.1038/76907.

3. Lee HY, Topham DJ, Park SY, Hollenbaugh J, Treanor J, Mosmann TR, et al. Simulation and prediction of the adaptive immune response to influenza A virus infection. *J Virol* (2009) **83(14):**7151–7165. doi:10.1128/JVI.00098-09.

4. Zarnitsyna VI, Handel A, McMaster SR, Hayward SL, Kohlmeier JE, Antia R. Mathematical model reveals the role of memory CD8 T cell populations in recall responses to influenza. *Front Immunol* (2016) **7:**165. doi:10.3389/fimmu.2016.00165.

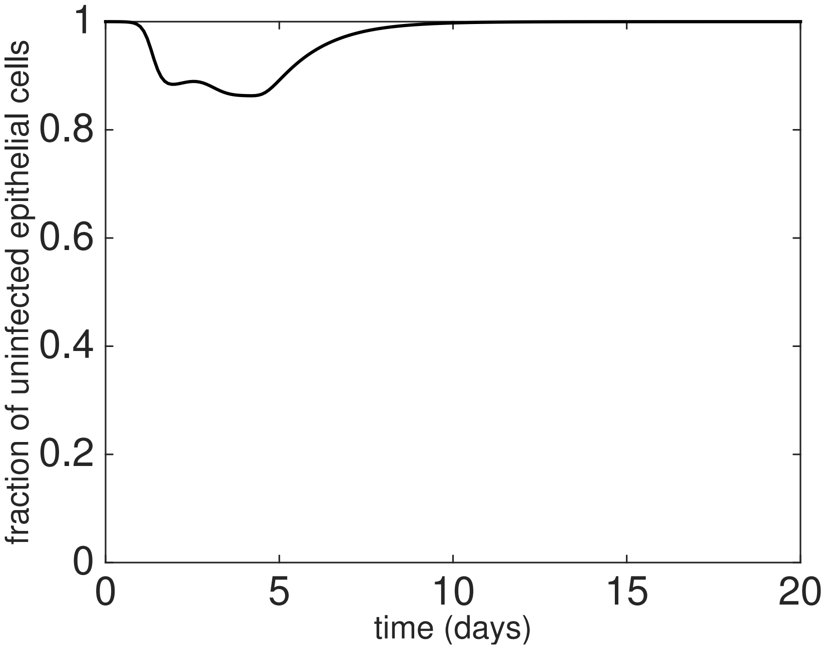

FIGURE S1: Model solution with parameters given in Table 1 in the main text shows that the loss of uninfected epithelial cells is maintained within 10–20% of total cell pool. This number of uninfected epithelial cells equals the sum of target cells T and resistant cells R shown in Fig. 3 in the main text.

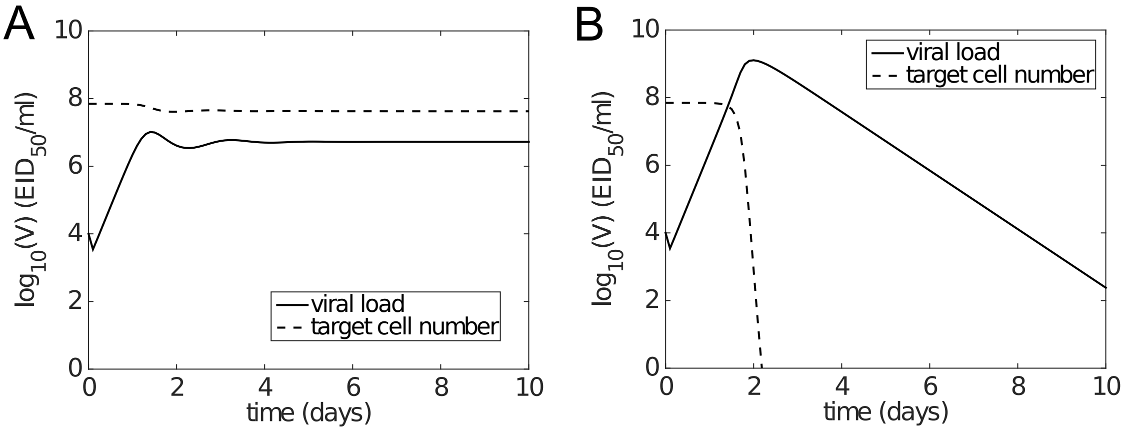

FIGURE S2: Inclusion of IFN prevents target cell depletion. In the absence of adaptive immunity (letting $C_n = B_n = 0$ in the model), the model with IFN production ($p_F = 10^{-5}$) simulates a sustained elevation of viral load and large steady state target cell number (dashed curve in panel **(A)**), both of which are consistent with previous experimental data (shown in Fig. 5 in the main text). However, in the absence of an innate response (letting $p_F = 0$), panel **(B)** shows that target cells are depleted, inducing a rapid fall in viral load. Note that for target cell number, the y-axis indicates $\log_{10}$(target cell number).

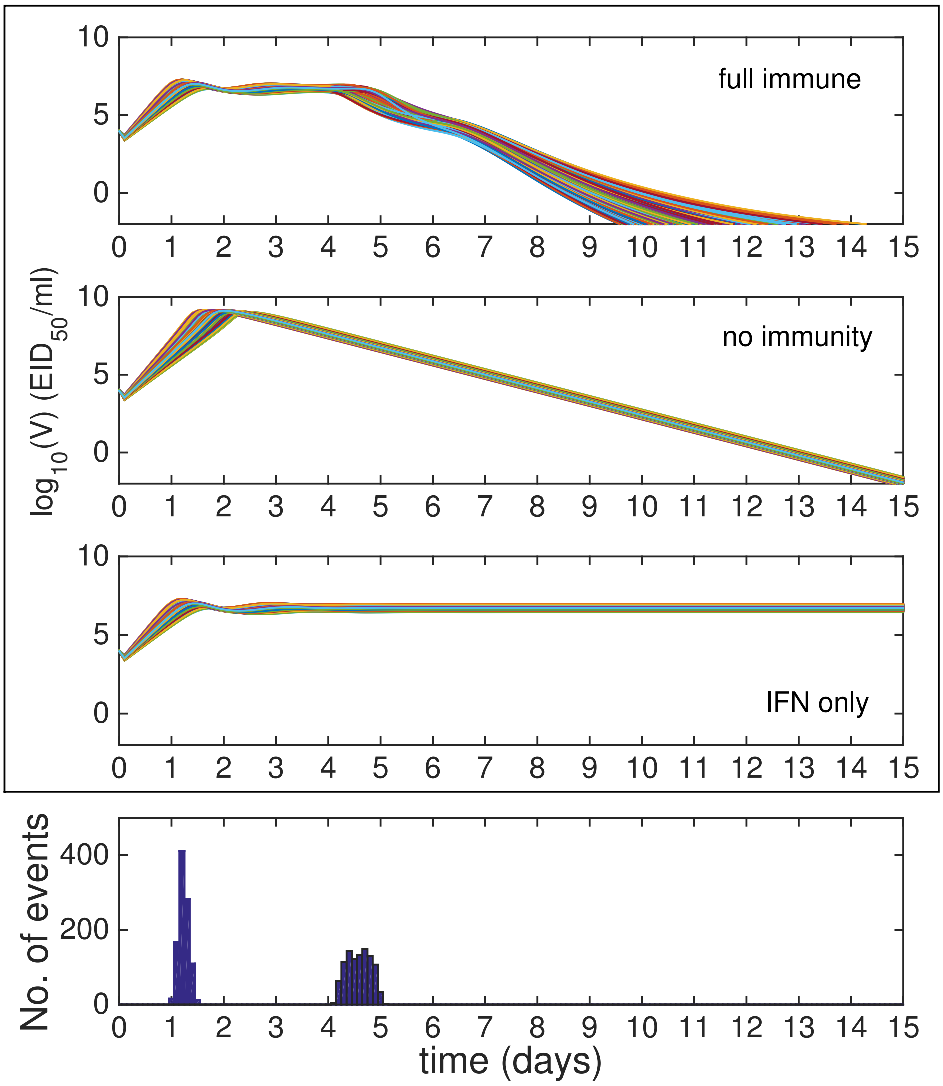

FIGURE S3: The three-phase model behavior is robust to the change of parameters. By allowing all the parameters to vary by ±20% from their default values (i.e. those shown in Table 1), 1000 samples of parameter sets were selected using Latin hypercube sampling and 1000 corresponding viral load solutions are shown in the upper panel. In Fig. 4 in the main text, we illustrated the phase separation using area shading. Here, the rough times of phase separation are indicated by the histograms in the lower panel; the left one indicates the time separating the first and the second phases (i.e. the time when the corresponding solutions of "full immune" and "no immunity" differ by 0.2 (in log-scale) for the first time) and right one indicates the time separating the second and the third phases (i.e. the time when the corresponding solutions of "full immune" and "IFN only" differ by 0.2 (in log-scale) for the first time). The solutions of "no immunity" are obtained by letting $p_F = 0$ and $\beta_{Cn} = \beta_{Bn} = 0$ in the model, and the solutions of "IFN only" are obtained by letting $\beta_{Cn} = \beta_{Bn} = 0$ in the model.

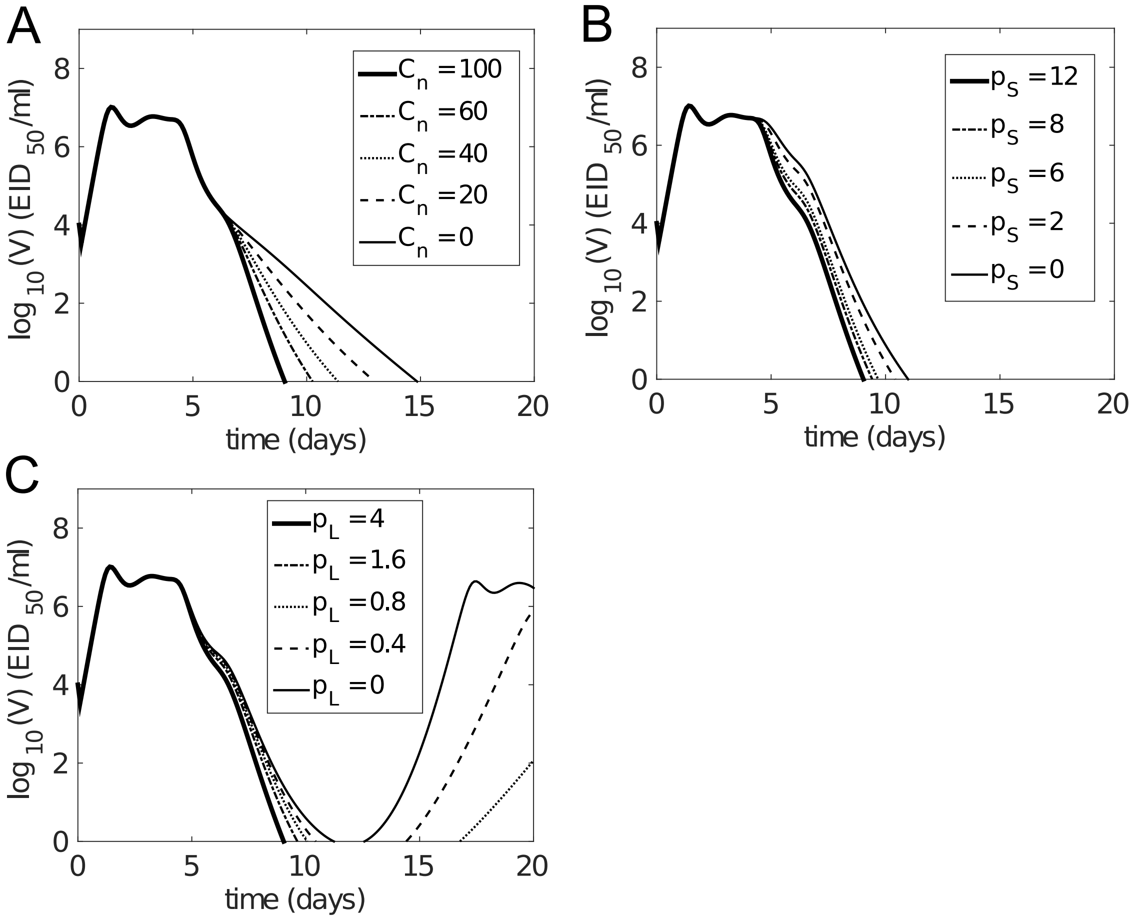

FIGURE S4: Dependence of model behaviour on the production rate of CD8$^+$ T cells (panel **(A)**), short-lived antibodies (panel **(B)**) and long-lived antibodies (panel **(C)**). Different levels of production for CD8$^+$ T cells, short-lived and long-lived antibodies are modelled respectively by varying the naive CD8$^+$ T cell number (C_n), short-lived antibody production rate (p_S) and long-lived antibody production rate (p_L). For each panel, all other model parameters were kept fixed at the values given in Table 1 in the main text.

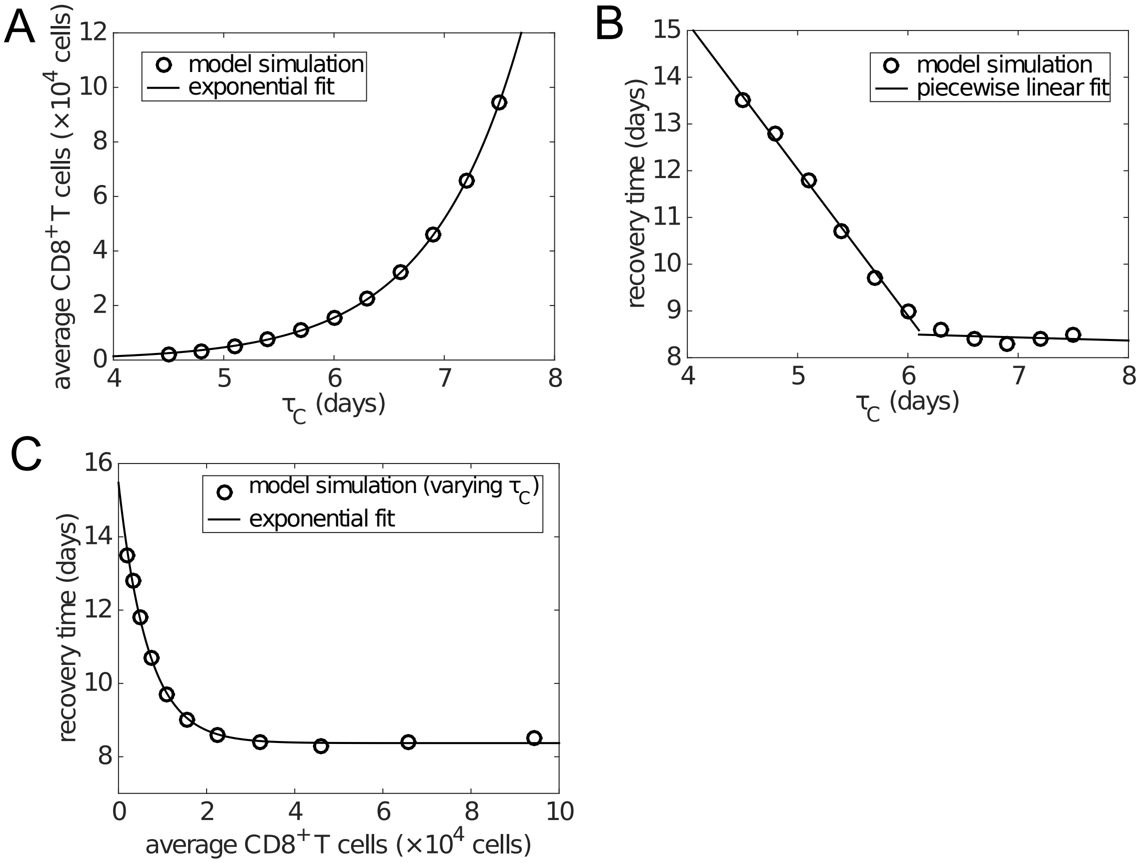

FIGURE S5: Dependence of the average effector CD8$^+$ T cell number (over days 6–20) on the delay induced by naive CD8$^+$ T cell activation and differentiation (τ_C). Recovery time is defined to be the time when viral load falls to 1 EID_{50}/ml. Panel **(A)** shows that the average effector CD8$^+$ T cell number is exponentially related to the delay τ_C. Panel **(B)** shows that the recovery time is related to the delay τ_C in an approximately piecewise linear manner. Panel **(C)** shows varying delay τ_C also preserves the approximately exponential relationship between the average CD8$^+$ T cell numbers and recovery time, consistent with the results of varying initial naive CD8$^+$ T cell numbers shown in Fig. 6 in the main text.

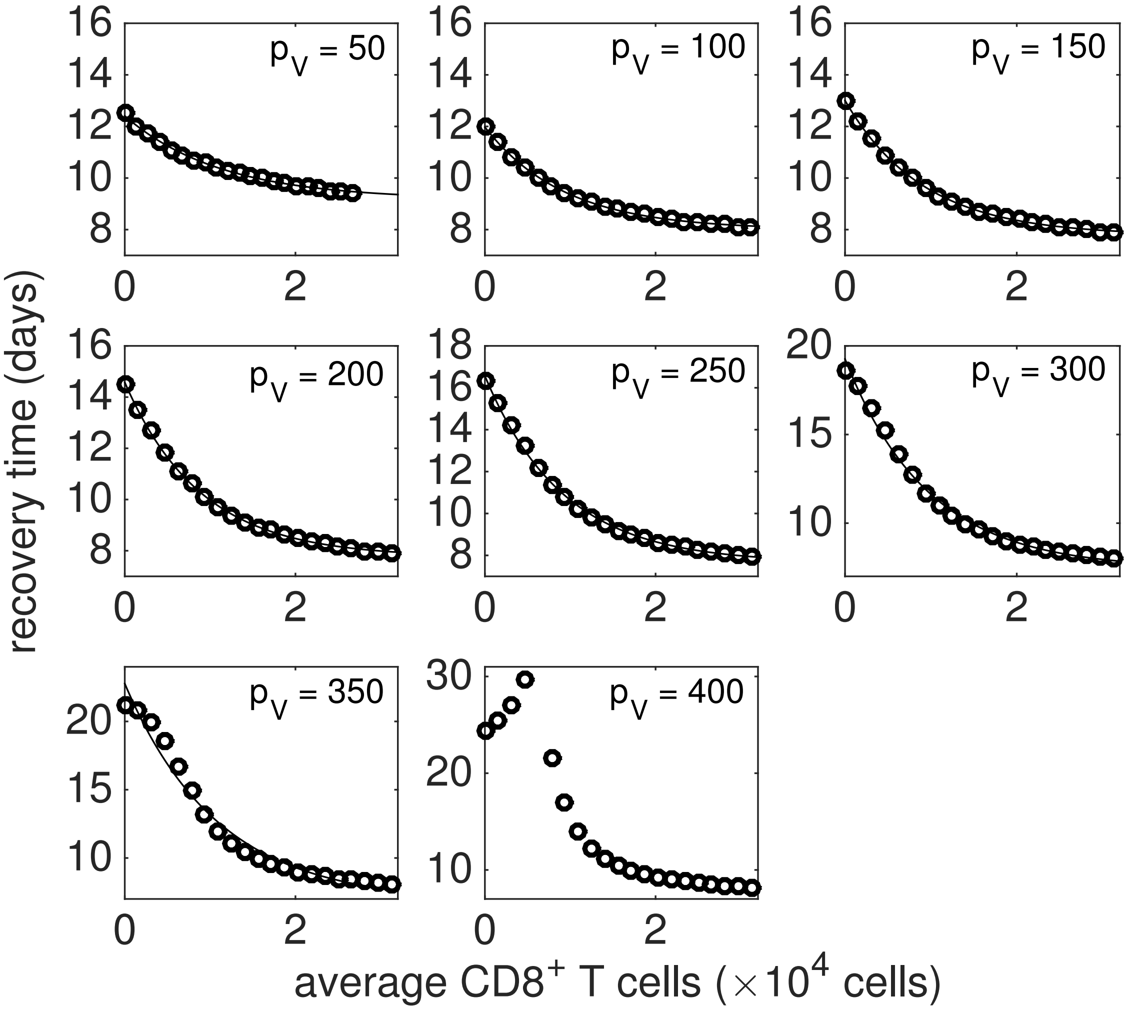

FIGURE S6: The exponential relationship between recovery time and the average effector CD8$^+$ T cell number (over days 6–20) is robust to the changes in the viral production rate p_V. Recovery time is defined to be the time when the viral load falls to 1 EID_{50}/ml. For each p_V, the relationship between average CD8$^+$ T cells and recovery time was obtained by varying initial naive CD8$^+$ T cell number. Except for large p_V (e.g. $p_V = 350, 400$), all other cases are well fit by exponential curves (thin black curves through the simulated data). When p_V is large (the cases of $p_V = 350, 400$), we observe a deviation from the exponential relationship for low expression of effector CD8$^+$ T cells. For example a decrease in recovery time is observed for a lower level of effector CD8$^+$ T cells (in the range of less than 5000 cells) for $p_V = 400$, whose reason is not clear and is likely a by-product of the highly complex dynamical system.

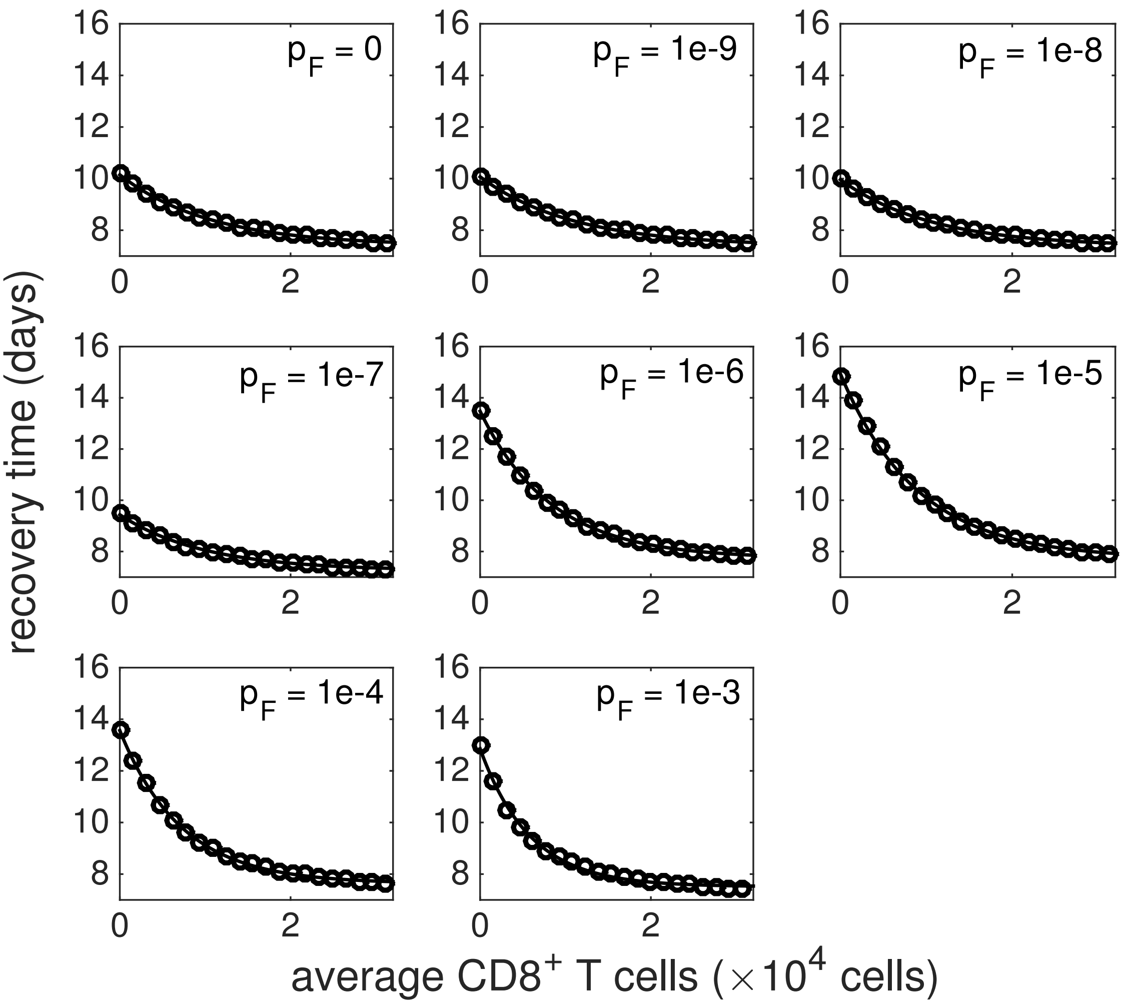

FIGURE S7: The exponential relationship between recovery time and the average effector $CD8^+$ T cell number (over days 6–20) is robust to the change of IFN production rate p_F. Recovery time is defined to be the time when viral load falls to 1 EID_{50}/ml. The thin black curves through the simulated data are exponential fits. One may observe that the recovery time increases and then decreases as p_F increases. The reason is not entirely clear. Our explanation is that the increase for small pF is likely because increasing p_F reduces the extent of target cell depletion and thus makes infection longer. When pF is sufficiently large, a further increase in p_F has little effect on target cell pool but induces stronger innate immune response, which play a role in shortening the recovery time.

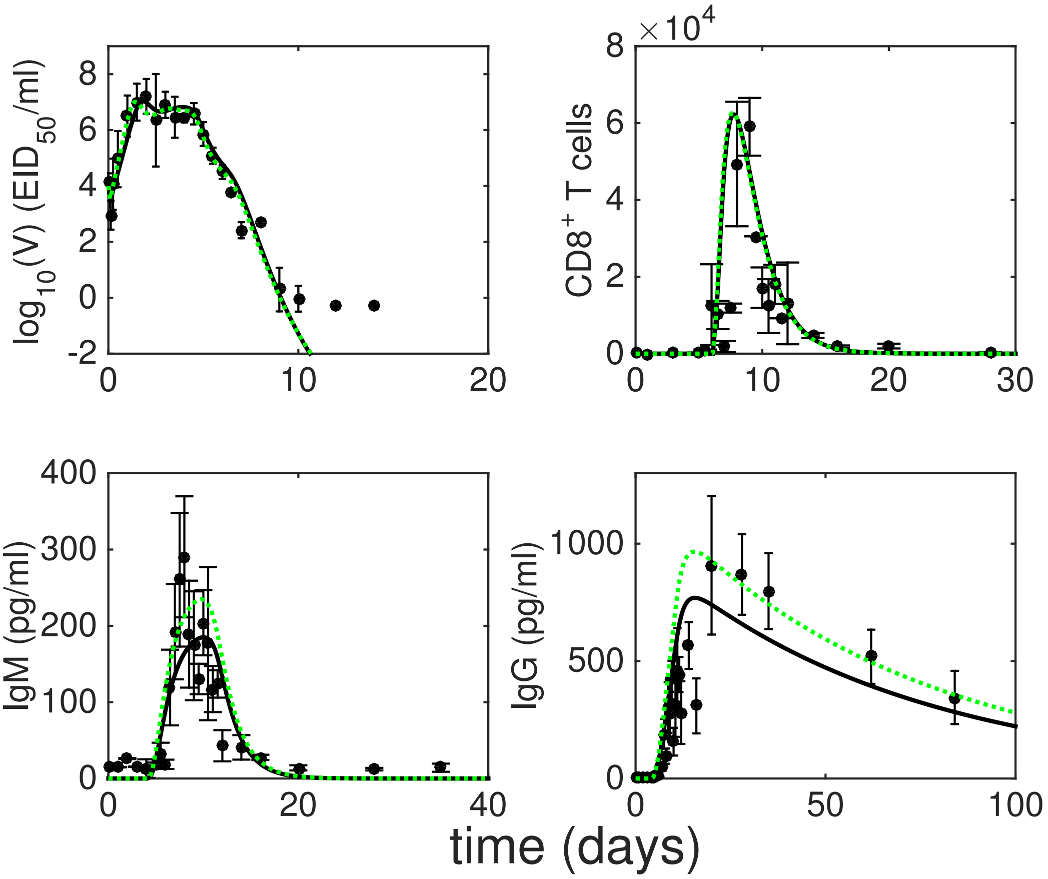

FIGURE S8: An example of a best-fit solution failing to capture the sparse part of the IgG data very well. Black dots are experimental data from (1) and black curves are the fits. The fit was generated by using equally weighted LSE function (scale factors remain unchanged) and the parameters in Table 1 in the main text as an initial guess in MATLAB's *fmincon* function. Compared to using weighted LSE (which generates the fit shown by green dashed curves), using equally weighted LSE does not alter the fits to the viral load and $CD8^+$ T cell number but significant underestimates antibody levels. Note that due to the limit of detection for the viral load (occurring after 10 days post-infection as seen in viral load data), the last three data points in the upper-left panel were not taken into consideration for model fitting.

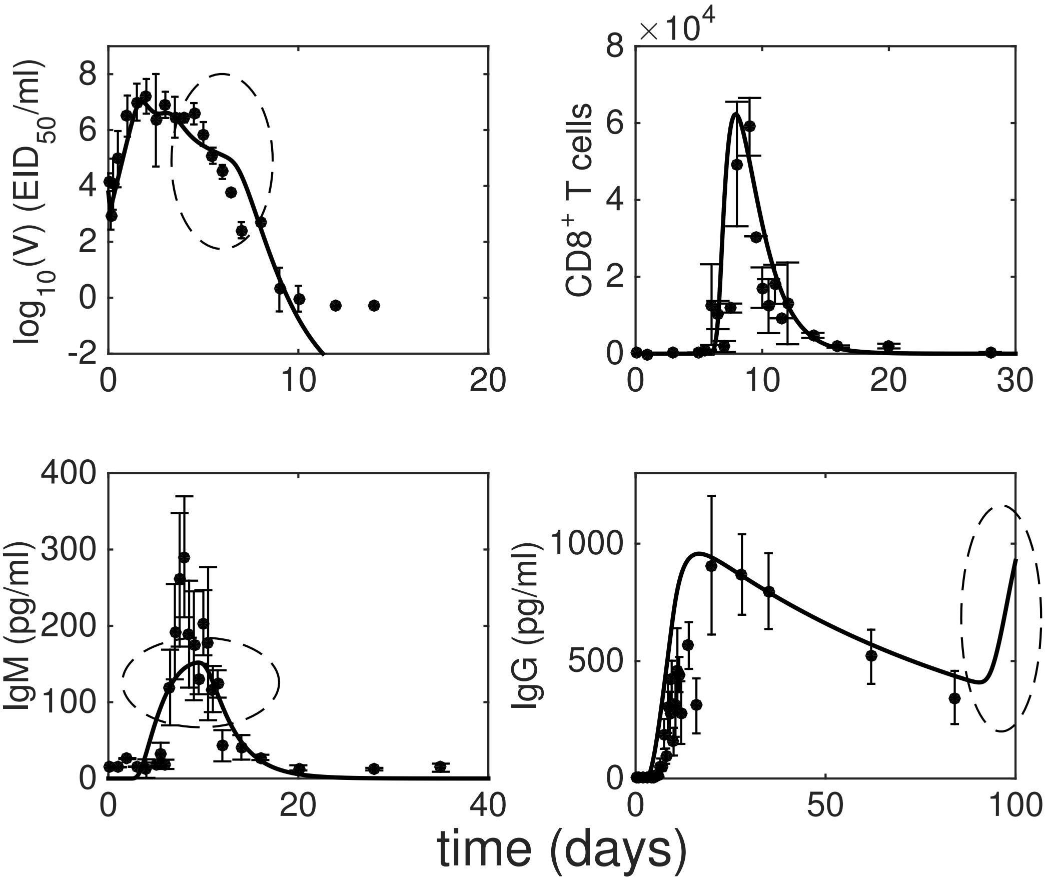

FIGURE S9: An example of a best-fit solution exhibiting oscillatory IgG, which was deemed biologically implausible. Black dots are experimental data from (1) and black curves are the fits. In fact, the LSE for this fit is smaller than that generated by the estimates in Table 1 in the main text. But given the fact that a few aspects of data were not well captured by this fit (indicated by dashed ovals), we did not consider this fit as an acceptable solution. Note that due to the limit of detection for the viral load (occurring after 10 days post-infection as seen in viral load data), the last three data points in the upper-left panel were not taken into consideration for model fitting.
